# Polyketide Derivatives from the Mangrove-Derived Fungus *Penicillium* sp. HDN15-312

**DOI:** 10.3390/md22080360

**Published:** 2024-08-08

**Authors:** Fuhao Liu, Wenxue Wang, Feifei Wang, Luning Zhou, Guangyuan Luo, Guojian Zhang, Tianjiao Zhu, Qian Che, Dehai Li

**Affiliations:** 1 Key Laboratory of Marine Drugs, Chinese Ministry of Education, School of Medicine and Pharmacy, Ocean University of China, Qingdao 266003, China; 17863905950@163.com (F.L.); bx_wwx@163.com (W.W.); wffxs2807@163.com (F.W.); 18895692529@163.com (L.Z.); luogy331@stu.ouc.edu.cn (G.L.); zhangguojian@ouc.edu.cn (G.Z.); 2 Laboratory for Marine Drugs and Bioproducts, Qingdao Marine Science and Technology Center, Qingdao 266237, China; 3 Sanya Oceanographic Institute, Ocean University of China, Sanya 572025, China

**Keywords:** OSMAC, *Penicillium* sp., DPPH, polyketides, theoretical calculations

## Abstract

Four new polyketides, namely furantides A–B (**1**–**2**), talamin E (**3**) and arugosinacid A (**4**), and two known polyketides were obtained from the mangrove-derived fungus *Penicillium* sp. HDN15-312 using the One Strain Many Compounds (OSMAC) strategy. Their chemical structures, including configurations, were elucidated by detailed analysis of extensive NMR spectra, HRESIMS and ECD. The DPPH radicals scavenging activity of **3**, with an IC_50_ value of 6.79 µM, was better than vitamin C.

## 1. Introduction

ROS (reactive oxygen species) play a twofold role as both toxic and beneficial compounds to organism’s systems. At optimal concentrations, ROS contribute positively to a range of physiological functions. They are integral to maintaining cellular homeostasis through redox regulation, modulating cell signaling pathways, and bolstering immune responses. However, when ROS levels escalate, their reactivity can become detrimental, leading to a state of oxidative stress [1,2]. Excessive ROS accumulation causes oxidative damage to mitochondria, lipids, proteins, RNA, and DNA [3,4]. Furthermore, oxidative stress also plays a key role in mediating inflammation, cardiovascular diseases, and the development of multiple cancers [5,6,7,8,9]. Antioxidants function by inhibiting or delaying the oxidation of chemicals [10]. Antioxidants exert their protective effects through two principal modes of action: The initial mechanism is characterized by chain breaking, wherein primary antioxidants sacrifice an electron to neutralize free radicals. The subsequent mechanism involves the intervention of secondary antioxidants, which eliminate the reactive species initiators by deactivating the catalysts that trigger the chain reaction [3]. At present, the antioxidants on the market can be divided into synthetic antioxidants (such as BHA, BHT, PG, etc.) and natural antioxidants (such as tea polyphenols, phytic acid, etc.) according to their sources [11]. Most of the antioxidants from natural sources are derived from terrestrial plants; there are also some lead compounds of marine origin with antioxidant activity [10], but no marketed antioxidant from secondary metabolites of marine fungal origin.

**Figure 1:**
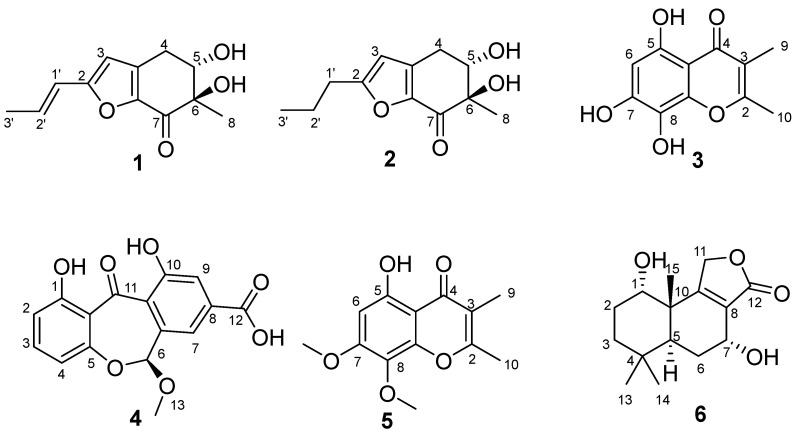
Structures of the isolated compounds **1**–**6**.

Marine organisms have been proven to be rich sources of structurally novel and biologically active secondary metabolites [12,13,14]. In the ongoing process of our group’s research for natural products with excellent bioactivity from marine-derived microorganisms, mangrove-derived fungus *Penicillium* sp. HDN15-312 has piqued our interest. The OSMAC strategy was employed for cultivating *Penicillium* sp. HDN15-312, which led to producing compounds with significantly different UV absorption profiles in modified Fungus 2# liquid media under static conditions, compared to other conditions. Under the guidance of HPLC-UV and LC-MS, four new compounds (**1**–**4**) and two known compounds (**5** and **6**) were obtained (Figure 1). Compounds **1**–**6** were evaluated for scavenging activity against DPPH radicals. The DPPH radical scavenging activity of **3**, with an IC_50_ value of 6.79 µM, was better than that of a natural antioxidant, vitamin C. Herein, we cover the isolation, structure elucidation, and DPPH scavenging activities of **1**–**6** from the mangrove-derived fungus *Penicillium* sp. HDN15-312.

## 2. Results

The *Penicillium* sp. HDN15-312 was meticulously isolated from a mangrove forest sample collected in Sanya, Hainan. A comprehensive study of the metabolites produced by this fungal species was conducted across six distinct culture media. Notably, the ethyl acetate extract derived from *Penicillium* sp. HDN15-312, when cultured in the modified Fungus 2# liquid medium, exhibited markedly distinct UV absorption profiles. Following these intriguing findings, a large-scale fermentation process was initiated, utilizing the same modified Fungus 2# liquid medium to optimize the production of the desired metabolites. As a result, the ethyl acetate extract was fractionated by extensive column chromatography (silica gel, LH-20, and HPLC) to obtain four undescribed compounds, named furantides A–B (**1**–**2**), talamin E (**3**) and arugosinacid A (**4**) (Figure 1).

**Figure 2:**
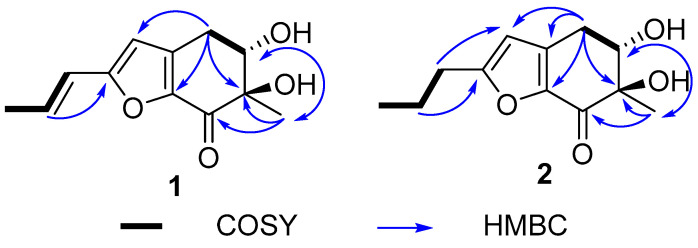
The key HMBC and COSY correlations in **1**–**2**.

Furantide A (**1**) was isolated as a white solid. High-resolution electrospray ionization mass spectrometry (HRESIMS) data provided the molecular formula C_12_H_14_O_4_ determined from the negative ion peak at *m*/*z* 223.0965 [M + H]^+^ (calcd for C_12_H_15_O_4_, 223.0965), indicating six degrees of unsaturation. The data of the ^1^H NMR, ^13^C NMR, (Table 1) and HSQC spectra of **1** showed 12 carbon signals, including two methyls, one methylene, four methines, four quaternary carbons, and one carbonyl carbon (*δ_C_* 188.7). The 1D NMR data of **1** (Table 1) were similar to aspergifuranone [15]. The difference was the replacement of a moiety of orsellinic acid at C-6 in aspergifuranone [15] by a hydroxyl group, which was supported by the NMR data (Figure 2) and a difference in molecular weight.

**Figure 3:**
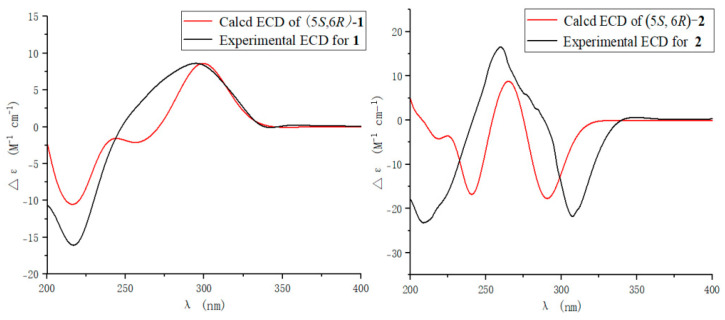
ECD spectra of **1** and **2**.

The side chain in **1** was identified as *trans*-unsaturated according to coupling constant of H-2′ (*J* = 15.8). In the ^1^H NMR spectrum, H-5 (*δ*_H_ 4.01) showed ax/eq coupling (*J* = 5.2 Hz) to H-4a (*δ*_H_ 3.07*)* and ax/ax coupling (*J* = 9.2 Hz) to H-4b (*δ*_H_ 2.66). In the NOE spectrum, upon irradiation of H_3_-8 (*δ*_H_ 1.31), a pronounced increase in the signal intensity from H-4b was observed (Figure S8), which implied that the methyl group and H-4b were oriented in the same spatial direction. In order to provide additional confirmation, we performed computational analysis of the NMR data, thereby determining the relative configurations. There were two relative configurations, named **1a** (5S*, 6R*) and **1b** (5S*, 6S*), theoretically. The two possible relative configurations of **1** were calculated by employing Time-Dependent Density Functional Theory (TDDFT) at the B3LYP/6-31+G(d)//B3LYP/6-311+G (d, p) levels. The result showed that **1a** (5S*, 6R*) represented a striking predominance (100% probability). (Table 2 and Figure S8) The absolute configuration of **1** was identified by ECD calculations at the B3LYP/6-31+G(d) level. Analysis of the ECD curve of **1** permitted us to come to the conclusion that the negative Cotton effect around 217 nm and positive Cotton effect around 298 nm indicated the 5*S* and 6*R* absolute configuration of **1** (Figure 3).

Furantide B (**2**) was isolated as a white solid. HRESIMS data provided the molecular formula C_12_H_16_O_4_, determined from the positive ion peak at *m*/*z* 225.1124 [M + H]^+^ (calcd for C_12_H_17_O_4_, 225.1121). NMR analysis revealed that the *trans*-unsaturated side chain double bonds (*δ*_C_ 133.6, 120.3) present in compound **1** were reduced to alkanes (*δ*_C_ 20.5, 29.6) in compound **2**. And, the absolute configuration of **2** was also the same as **1,** which was determined by calculating NMR data and ECD (Table 2, Figure 3 and Figure S16).

**Figure 4:**
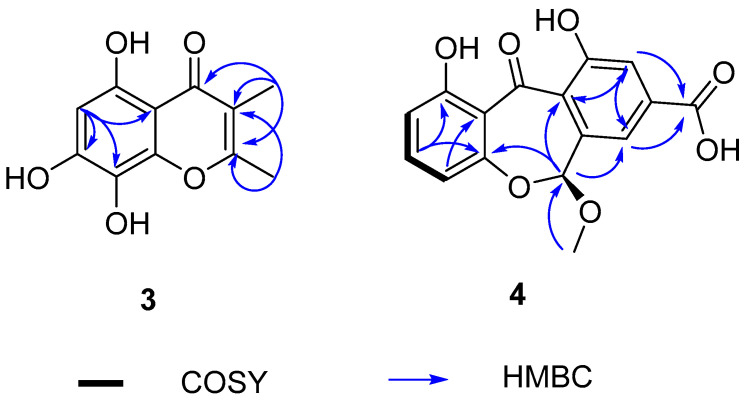
The key HMBC correlations of **3** and **4**.

Talamin E (**3**) was isolated as a pale yellow powder with the molecular formula of C_11_H_10_O_5_ on the basis of HRESIMS data at *m/z* 223.0595 [M + H]^+^ (calcd for C_11_H_11_O_5_, 223.0601), indicating seven degrees of unsaturation. The ^1^H and ^13^C NMR data (Table 3) showed two singlet methyls, eight olefinic carbons (*δ_C_* 99.2, 104.2, 115.2, 125.7, 146.7, 153.7, 154.9 and 164.6) and one carbonyl carbon (*δ*_C_ 183.5). Discreet analysis of the NMR data indicated that **3** and talamin B [16] (**5**) shared the same skeleton. The key distinction was the substitution of two methoxy groups at the C-7, C-8 position respectively in **5** with hydroxyl groups, which was corroborated by the absence of HSQC correlation for the methyl group and the molecular formula (Table 3 and Figure 4). Thus, compound **3** was determined as 5,7,8-dihydroxy-2,3-dimethylchromone, referred to as talamin E.

Arugosinacid A (**4**), isolated as yellow powder, had a molecular formula of C_16_H_12_O_7_ from the negative ion peak at *m/z* [M − H]^−^ 315.0505 (calcd for C_16_H_11_O_7_, 315.0501) in the HRESIMS spectrum, indicating 11 degrees of unsaturation. Analysis of ^1^H NMR, ^13^C NMR and HSQC data (Table 4 and Figure 4) revealed the presence of 16 carbons, including one methoxy group, twelve aromatic carbons, one methine carbon, and two possible carbonyl carbons (*δ*_C_ 194.8, 166.2), which shared a similar skeletal structure with arugosin K [17]. The differences were the absence of isopentenyl at C-2 supported by HSQC correlation from H-2 (*δ*_H_ 6.67) to C-2 (*δ*_C_ 110.5) and ^1^H-^1^H COSY of H-2 to H-3 (*δ*_H_ 7.50), along with the replacement of methyl at C-8 (*δ*_C_ 134.8) by a carboxyl group (*δ_C_* 166.2), which was manifested in HMBC correlation from H-7 (*δ*_H_ 7.60) and H-9 (*δ*_H_ 7.56) to C-12 (*δ*_C_ 166.2). Based on these findings, the planar structure was successfully elucidated. It can be known from the literature [18] that the planar structure of this compound has appeared in an article on synthesis, but the stereostructure has not been characterized.

**Figure 5:**
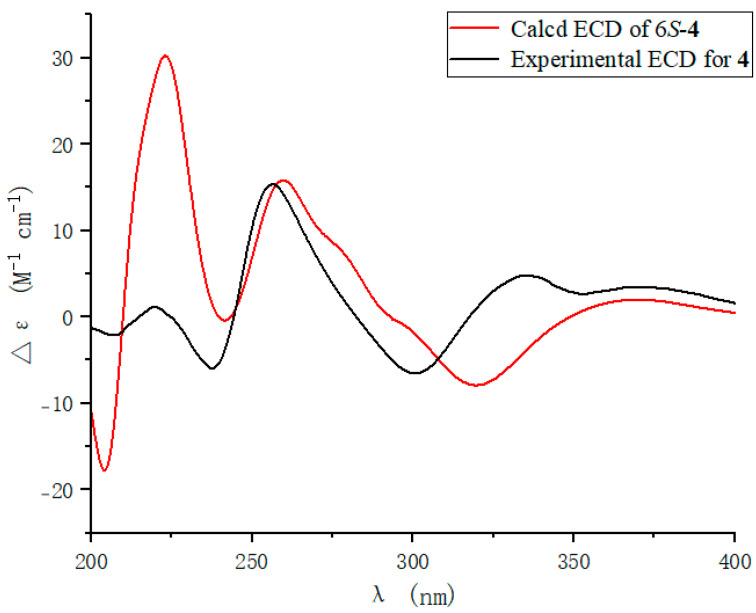
ECD spectra of **4**.

To further determine the absolute configuration of **4**, the optimized conformations of (6*S*)-**4** were obtained at the B3LYP/6-31+G(d) level and used for ECD calculations. The agreement of the experimental and calculated ECD curves (Figure 5) indicated the 6*S* absolute configuration of **4.**

Compound **5** was isolated as a pale yellow powder. The ^1^H and ^13^C NMR data (Table 3) showed two singlet methyls (*δ_C_* 9.1, 18.5), two methoxy groups (*δ_C_* 56.9, 61.8), eight olefinic carbons (*δ_C_* 96.3, 104.8, 115.7, 129.6, 150.7, 158.5, 159.6, 165.0) and one carbonyl carbon (*δ*_C_ 183.5). Upon careful analysis of the ^1^H NMR, ^13^C NMR, and HMBC spectrum (Table 3 and Table S1 and Figures S36–S39), it was found that compound **5** was identical to the known talamin B [16]. Therefore, compound 5 was identified as talamin B.

Compound **6** was isolated as a white solid and was identified astalaminoid C [19] through meticulous comparison of its physical and spectroscopic characteristics with those documented in the existing literature (Table S2, Figures S40 and S41).

Compounds **1**–**6** were evaluated for 2,2-diphenyl-1-picrylhydrazyl (DPPH) radical eliminating activity. Consequently, **3**, **4,** and **6** had DPPH radical eliminating activities, with IC_50_ values of 6.79 to 56.92 µM (Table 5). Among them, **3** displayed significantly better values than those of the positive control Vitamin C (IC_50_ = 12.15 µM). Analysis of compounds **3** and **5** shows that the hydroxyl group is the basis for antioxidant activity, and the hydroxyl group at position C-8 of **3** may be essential for bioactivity.

## 3. Materials and Methods

### 3.1. General Experimental Procedures

The HRESIMS data were measured on a Thermo Scientific LTQ Orbitrap XL mass spectrometer (Thermo Fisher Scientific, Waltham, MA, USA). UV spectra were recorded on a Hitachi 5430 spectrophotometer (Hitachi Ltd., Tokyo, Japan). NMR spectra were collected on Bruker AVANCE NEO 400 MHz spectrometer and JEOL JNM-ECZ600R/S1 spectrometer with tetramethylsilane (TMS) as an internal standard. Column chromatography (CC) was performed with silica gel (300–400 mesh, Qingdao Marine Chemical Inc., Qingdao, China) and Sephadex LH-20 (Amersham Biosciences, Buckinghamshire, UK). MPLC was performed on a Waters 1526. HPLC spectra were recorded on a Hitachi Primaide HPLC using an ODS column (HPLC (YMC-Pack ODS-A, 10 × 250 mm, 5 µm, 3 mL/min)) (YMC Co., Ltd., NJ, USA).

### 3.2. Fungi Material and Fermentation

*Penicillium* sp. HDN15-312 (GenBank No.MK41874) was isolated from a sample of a mangrove forest in Sanya, Hainan. It was deposited at Key Laboratory of Marine Drugs, the Ministry of Education of China, School of Medicine and Pharmacy, Ocean University of China, Qingdao, People’s Republic of China. This strain is a facultative anaerobe, producing an aerial mycelium that is beige to light yellow, a substrate mycelium that is green, and straight to curved hyphae with long, straight spore chains.

### 3.3. OSMAC Study, Fermentation, and Extraction

Based on the OSMAC strategy, six different liquid media, labeled as Fungus 2# liquid media (glucose 1%, maltose 2%, mannitol 2%, monosodium glutamate 1%, KH_2_PO_4_ 0.05%, MgSO_4_·7H_2_O 0.03%, corn steep liquor 0.1% and yeast extract 0.3%, seawater), modified Fungus 2# liquid media (soluble starch 4%, yeast extract 0.1%, MgSO_4_·7H_2_O 0.03%, monosodium glutamate 0.2%, sucrose 4%, KH_2_PO_4_ 0.05%, maltose 3%, soybean flour 0.05%, peptone 0.2%, seawater), SDA liquid media (peptone 1%, NaCl 0.5%, glucose 4%, freshwater), oligotrophic liquid media (soluble starch 1%, peptone 0.1%, seawater), PDB liquid media (glucose 20 g/L, potato extract 200 g/L, seawater), and glycerol liquid media (glycerin 2%, peptone 0.2%, yeast powder 0.4%, seawater) were utilized to culture the strain HDN15-312 under static or shaking conditions. The products of the strain HDN15-312 cultured in modified Fungus 2# liquid media under static conditions were different from the products under other conditions. Therefore, the modified Fungus 2# liquid media under static conditions was selected as the optimal condition for large-scale fermentation. The fungus was cultured in 1000 mL Erlenmeyer flasks, each containing 300 mL of modified Fungus 2# liquid media, at 28 °C for 30 days after adjusting its pH to 6.5 in natural seawater (collected from JiaoZhou Bay, Qingdao, China). A total of 15 L of broth was extracted using EtOAc (3 × 15 L) to obtain an extract weighing 50 g.

### 3.4. Isolation and Purification of Compounds

The crude extract was applied over a VLC column and eluted with mixtures of Petroleum ether-EtOAc to give nine fractions (Fr.1–Fr.9). Fr.2–Fr.5 were combined as Fr.A, which was separated by MPLC using an ODS column to obtain ten subfractions (Fr.A.1–Fr.A.10). Fr.A.6 was purified by semi-preparative HPLC to obtain **1** (3 mg, *t*_R_ = 15 min) and **2** (2 mg, *t*_R_ = 16 min). Fr.A.7 was purified by semi-preparative HPLC to obtain **3** (2.5 mg, *t*_R_ = 11 min). Fr.6–Fr.8 were combined as Fr.B, which was separated by HPLC using an ODS column to obtain ten subfractions (Fr.B.1–Fr.B.8). Fr.B.6 was purified by semi-preparative HPLC to obtain **4** (3 mg, *t*_R_ = 15 min) and **5** (5 mg, *t*_R_ = 19 min). Fr.B.3 was purified by semi-preparative HPLC to obtain **6** (3 mg, *t*_R_ = 11 min).

Furantide A (**1**): pale yellow powder, ${\left[\alpha \right]}_{D}^{25}$ +45.6 (*c* 0.05 MeOH); UV (MeOH) λ_max_ 217 (1.8), 322 (1.3) nm; IR (KBr) *ν*_max_ 3298, 2932, 1671, 1446 cm^−1^; ^1^H and ^13^C NMR data, Table 1; HRESIMS *m*/*z* 223.0965 [M + H]^+^ (calcd for C_12_H_15_O_4_, 223.0965).

Furantide B (**2**): pale yellow powder, ${\left[\alpha \right]}_{D}^{25}$ +81.4 (*c* 0.1 MeOH); UV (MeOH) λ_max_ 206 (0.9), 234 (0.8), 297 (1.9) nm; IR (KBr) *ν*_max_ 2967, 2873, 1671, 1528, 1447 cm^−1^; ^1^H and ^13^C NMR data, Table 1; HRESIMS *m*/*z* 225.1124 [M + H]^+^ (calcd for C_12_H_17_O_4_, 225.1121).

Talamin E (**3**): yellow powder, ${\left[\alpha \right]}_{D}^{25}$ +56.2 (*c* 0.05 MeOH); UV (MeOH) *λ*_max_ 226 (0.3), 260 (0.6), 344 (0.1) nm; IR (KBr) *ν*_max_ 1655, 1576, 1523, 1436, 1198 cm^−1^; ^1^H and ^13^C NMR data, Table 2; HRESIMS *m*/*z* 223.0595 [M + H]^+^ (calcd for C_11_H_11_O_5_, 223.0601).

Arugosinacid A (**4**): yellow powder, ${\left[\alpha \right]}_{D}^{25}$ −37.5 (*c* 0.05 MeOH); UV (MeOH) *λ*_max_ 216 (0.5), 263 (0.4), 344 (0.1) nm; IR (KBr) *ν*_max_ 3736.19, 2932.84, 1682.38, 1586.14, 1206.97 cm^−1^; ^1^H and ^13^C NMR data, Table 2; HRESIMS *m*/*z* 315.0505 [M − H]^−^ (calcd for C_16_H_11_O_7_, 315.0501).

### 3.5. Computation Section

Conformational searches were conducted using Spartan’14 [20], based on the MMFF (Merck Molecular Force Field). Compounds **1**, **2**, and **4** were further optimized with DFT calculations at the B3LYP/6-31+G(d) level, utilizing the Gaussian 09 program [21]. TDDFT calculations were performed on the two lowest-energy conformations for **1** and **2** (>5% population). ECD spectra were gained using the SpecDis program [22] in conjunction with a Gaussian [21] band shape with 0.2 eV width for **1**, 0.4 eV width for **2**, and 0.1 eV width for **4** from dipole-length rotational strengths. The calculated spectra were shifted by −10 nm for **1**, −20 nm for **2**, +5 nm for **4** to facilitate comparison to the experimental data. We acknowledge the support of the High-Performance Biological Supercomputing Center at the Ocean University of China for this research.

### 3.6. Assay of DPPH Activity

Based on the method of Sharma [23], with some amendments, the eliminating activity against DPPH radicals was enforced. The compounds **1**–**6** and vitamin C were dissolved in absolute ethanol and diluted into 5 gradients. These were vortexed to mix well, protected from light at room temperature for 30 min, and the absorbance was read at 515 nm as a positive control.

The ability to scavenge the DPPH was calculated according to the equation:

DPPH free radical scavenging rate D VC% = [(A_blank_ − A_control_) ÷ A_blank_] × 100%

DPPH free radical scavenging rate D _sample_% = [[A_blank_ − (A_sample_ − A_control_)] ÷ A_blank_] × 100%

A_control_: the absorbance of the DPPH solution;

A_blank_: the absorbance of ethanol.

## 4. Conclusions

In conclusion, four polyketides (**1**–**4**) were isolated and determined from *Penicillium* sp. HDN15-312, which was isolated from a sample of a mangrove forest in Egret Park in Sanya, Hainan. Compounds **1** and **2** stood as the unique pair of naturally occurring substances characterized by their distinctive 6-methyl-cyclohexanone-furan ring structure. The bioactivity screening showed that compounds **3**, **4** and **6** showed potent DPPH radical scavenging capacities. The DPPH radicals scavenging activity of **3,** with an IC_50_ value of 6.79 µM, was better than vitamin C. Our research highlights the potential for sifting and exploiting therapeutic molecules from marine fungi.

## Figures and Tables

**Table 1:** NMR data of **1** in CD_3_OD-*d*_4_ and **2** in DMSO-*d*_6_ (*δ* in ppm, 400 MHz for ^1^H and 100 MHz for ^13^C).

No.	1		2	
	*δ*_H_, (*J* in Hz)	*δ*_C_, Type	*δ*_H_, (*J* in Hz)	*δ*_C_, Type
2		161.4, C		163.3, C
3	6.35, s	109.0, CH	6.28, s	108.4, CH
3a		140.4, C		137.4, C
4	4a: 3.07, dd (16.9, 5.2), 4b: 2.66, dd (16.9, 9.2)	29.6, CH_2_	4a: 2.97, dd (16.9, 4.5) 4b: 2.52, m	28.4, CH_2_
5	4.01, dd (9.2, 5.2)	75.2, CH	3.85, dt (7.3, 4.5)	73.8, CH
6		79.2, C		76.9, C
7		188.7, C		186.2, C
7a		145.5, C		144.4, C
8	1.31, s	18.4, CH_3_	1.18, s	18.7, CH_3_
1′	6.36, dd (15.8, 1.7)	120.3, CH	2.64, t (7.5)	29.6, CH_2_
2′	6.58, dd (15.8, 6.8)	133.6, CH	1.63, m	20.5, CH_2_
3′	1.91, dd (6.9, 1.7)	18.7, CH_3_	0.91, t (7.4)	13.6, CH_3_

**Table 2:** DP4+ computational analysis of the relative configurations of **1** and **2**.

DP4+	1		2	
	1a	1b	2a	2b
^1^H	100.00%	0.00%	100.00%	0.00%
^13^C	100.00%	0.00%	100.00%	0.00%
^1^H + ^13^C	100.00%	0.00%	100.00%	0.00%

**Table 3:** NMR data of compound **3** and **5** in CD_3_OD-*d*_4_ (*δ* in ppm, 400 MHz for ^1^H and 150 MHz for ^13^C).

No.	3		5	
	*δ*_H_, (*J* in Hz)	*δ*_C_, Type	*δ*_H_, (*J* in Hz)	*δ*_C_, Type
2		164.6, C		165.0, C
3		115.2, C		115.7, C
4		183.5, C		183.5, C
4a		104.2, C		104.8, C
5		154.9, C		159.6, C
6	6.23, s	99.2, CH	6.47, s	96.3, CH
7		153.7, C		158.5, C
8		125.7, C		129.6, C
8a		146.7, C		150.7, C
9	1.98, d (0.8)	9.2, CH_3_	1.99, s	9.1, CH_3_
10	2.44, d (0.8)	18.4, CH_3_	2.45, s	18.5, CH_3_
11			3.91, s	61.8, CH_3_
12			3.81, s	56.9, CH_3_

**Table 4:** NMR data of compound **4** in DMSO-*d*_6_ (*δ* in ppm, 400 MHz for ^1^H and 150 MHz for ^13^C).

No.	4	
	*δ*_H_, (*J* in Hz)	*δ*_C_, Type
1		161.2, C
2	6.67, dd (8.2, 1.1)	110.5, CH
3	7.50, t (8.2)	137.9, CH
4	6.62, dd (8.2, 1.1)	109.9, CH
5		154.9, C
6	6.19, s	102.3, CH
7a		136.9, C
7	7.60, d (1.5)	115.3, CH
8		134.8, C
9	7.56, d (1.6)	118.7, CH
10		156.9, C
10a		126.1, C
11		194.7, C
11a		113.5, C
12		166.2, C
13	3.45, s	56.6, CH_3_

**Table 5:** DPPH radical scavenging activity of compounds **1**–**6** (IC_50_, µM).

Compd.	1	2	3	4	5	6	Vitamin C
IC_50_ (µM)	>200	>200	6.79	56.92	>200	32.11	12.15

## Data Availability

The data presented in this study are available in this article and Supplementary Material.

## Supplementary Materials

The following supporting information can be downloaded at: https://www.mdpi.com/article/10.3390/md22080360/s1, Figure S1: OSMAC strategy for cultivating Penicillium sp. HDN15-312; Figure S2. HRESIMS spectrum of compound 1; Figure S3. ^1^H NMR (400 MHz, MeOD-*d_4_*) spectrum of compound **1**; Figure S4. ^13^C NMR (100 MHz, MeOD-*d*_4_) of compound 1; Figure S5. COSY spectrum of compound **1**; Figure S6. HSQC spectrum of compound **1**; Figure S7. HMBC spectrum of compound **1**; Figure S8. NOE spectrum of compound 1; Figure S9. DP4+ computational analysis the relative configurations of 1; Figure S10. UV spectrum of compound **1**; Figure S11. IR spectrum of compound **1**; Figure S12. HRESIMS spectrum of compound **2**; Figure S13. ^1^H NMR (400 MHz, DMSO-*d_6_*) spectrum of compound **2**; Figure S14. ^13^C NMR (100 MHz, DMSO-*d_6_*) of compound **2**; Figure S15. COSY spectrum of compound **2**; Figure S16. HSQC spectrum of compound **2**; Figure S17. HMBC spectrum of compound **2**; Figure S18. DP4+ computational analysis the relative configurations of 2; Figure S19. UV spectrum of compound **2**; Figure S20. IR spectrum of compound **2**; Figure S21. HRESIMS spectrum of compound **3**; Figure S22. ^1^H NMR (400 MHz, MeOD-*d_4_*) spectrum of compound 3; Figure S23. ^13^C NMR (150 MHz, MeOD-*d_4_*) of compound **3**; Figure S24. HSQC spectrum of compound **3**; Figure S25. HMBC spectrum of compound **3**; Figure S26. UV spectrum of compound **3**; Figure S27. IR spectrum of compound **3**; Figure S28. HRESIMS spectrum of compound **4**; Figure S29. ^1^H NMR (400 MHz, DMSO-*d_6_*) spectrum of compound **4**; Figure S30. ^13^C NMR (150 MHz, DMSO-*d_6_*) spectra of compound **4**; Figure S31. COSY spectrum of compound **4**; Figure S32. HSQC spectrum of compound 4; Figure S33. HMBC spectrum of compound **4**; Figure S34. UV spectrum of compound **4**; Figure S35. IR spectrum of compound **4**; Figure S36. ^1^H NMR (400 MHz, MeOD-*d_4_*) spectrum of compound **5**; Figure S37. ^13^C NMR (150 MHz, MeOD-*d_4_*) spectra of compound **5**; Figure S38. ^1^H NMR (400 MHz, DMSO-*d_6_*) spectrum of compound **5**; Figure S39. ^13^C NMR (100 MHz, DMSO-*d_6_*) spectra of compound **5**; Figure S40. ^1^H NMR (400 MHz, MeOD-*d_4_*) spectrum of compound **6**; Figure S41.^13^C NMR (100 MHz, MeOD-*d_4_*) spectra of compound **6**. Table S1. The comparison of NMR data between compound **5** in DMSO-*d_6_* and talamin B in DMSO-*d_6_*; Table S2. The comparison of NMR data between compound **6** in CD_3_OD-*d_4_* and astalaminoid C in CDCl_3_-*d.*
