# Supplementary material for: Polyketide Derivatives from the Mangrove-Derived Fungus Penicillium sp. HDN15-312
Source: Mar Drugs. 2024 Aug 8;22(8):360. doi: 10.3390/md22080360 (PMC11355304; doi:10.3390/md22080360)
Supplement: Supplementary file 1 [file marinedrugs-22-00360-s001.zip › marinedrugs-3149236-supplementary.pdf]

Supplementary Material

Polyketide Derivatives from the Mangrove-Derived Fungus *Penicillium* sp. HDN15-312

Fuhao Liu [1,†], Wenxue Wang [1,†], Feifei Wang [1], Luning Zhou [1], Guangyuan Luo [1], Guojian Zhang [1,2], Tianjiao Zhu [1,*], Qian Che [1,*] and Dehai Li [1,2,3,*]

[1] Key Laboratory of Marine Drugs, Chinese Ministry of Education, School of Medicine and Pharmacy, Ocean University of China, Qingdao 266003, China; 17863905950@163.com (F.L.); bx_wwx@163.com (W.W.); wffxs2807@163.com (F.W.); 18895692529@163.com (L.Z.); luogy331@stu.ouc.edu.cn (G.L.); zhangguojian@ouc.edu.cn (G.Z.); zhutj@ouc.edu.cn (T.Z.)

[2] Laboratory for Marine Drugs and Bioproducts, Qingdao Marine Science and Technology Center, Qingdao 266237, China

[3] Sanya Oceanographic Institute, Ocean University of China, Sanya 572025, China

* Correspondence: Zhutj@ouc.edu.cn (T.Z.); cheqian064@ouc.edu.cn (Q.C.); dehaili@ouc.edu.cn (D.L.); Tel.: +86-532-82031619 (D.L.).

† These authors contributed equally to this work.

Contents

Figure S1. OSMAC strategy for cultivating *Penicillium sp*. HDN15-312;

Figure S2. HRESIMS spectrum of compound **1**;

Figure S3. ^{1}H NMR (400 MHz, MeOD-d_4) spectrum of compound **1**;

Figure S4. ^{13}C NMR (100 MHz, MeOD-d_4) of compound **1**;

Figure S5. COSY spectrum of compound **1**;

Figure S6. HSQC spectrum of compound **1**;

Figure S7. HMBC spectrum of compound **1**;

Figure S8. NOE spectrum of compound **1**;

Figure S9. The corresponding structure when DP4+ calculating the relative configuration of compound **1**;

Figure S10. UV spectrum of compound **1**;
Figure S11. IR spectrum of compound **1**;
Figure S12. HRESIMS spectrum of compound **2**;
Figure S13. ^{1}H NMR (400 MHz, DMSO-d_6) spectrum of compound **2**;
Figure S14. ^{13}C NMR (100 MHz, DMSO-d_6) of compound **2**;
Figure S15. COSY spectrum of compound **2**;
Figure S16. HSQC spectrum of compound **2**;
Figure S17. HMBC spectrum of compound **2**;
Figure S18. The corresponding structure when DP4+ calculating the relative configuration of compound **2**;
Figure S19. UV spectrum of compound **2**;
Figure S20. IR spectrum of compound **2**;
Figure S21. HRESIMS spectrum of compound **3**;
Figure S22. ^{1}H NMR (400 MHz, MeOD-d_4) spectrum of compound **3**;
Figure S23. ^{13}C NMR (150 MHz, MeOD-d_4) of compound **3**;
Figure S24. HSQC spectrum of compound **3**;
Figure S25. HMBC spectrum of compound **3**;
Figure S26. UV spectrum of compound **3**;
Figure S27. IR spectrum of compound **3**;
Figure S28. HRESIMS spectrum of compound **4**;
Figure S29. ^{1}H NMR (400 MHz, DMSO-d_6) spectrum of compound **4**;
Figure S30. ^{13}C NMR (150 MHz, DMSO-d_6) spectra of compound **4**;
Figure S31. COSY spectrum of compound **4**;
Figure S32. HSQC spectrum of compound **4**;
Figure S33. HMBC spectrum of compound **4**.
Figure S34. UV spectrum of compound **4**;
Figure S35. IR spectrum of compound **4**;

Figure S36. ^{1}H NMR (400 MHz, MeOD-d_4) spectrum of compound **5**;
Figure S37. ^{13}C NMR (150 MHz, MeOD-d_4) spectra of compound **5**;
Figure S38. ^{1}H NMR (400 MHz, DMSO-d_6) spectrum of compound **5**;
Figure S39. ^{13}C NMR (150 MHz, DMSO-d_6) spectra of compound **5**;
Figure S40. ^{1}H NMR (400 MHz, MeOD-d_4) spectrum of compound **6**;
Figure S41. ^{13}C NMR (150 MHz, MeOD-d_4) spectra of compound **6.**

Figure S1. OSMAC strategy for cultivating *Penicillium sp*. HDN15-312.

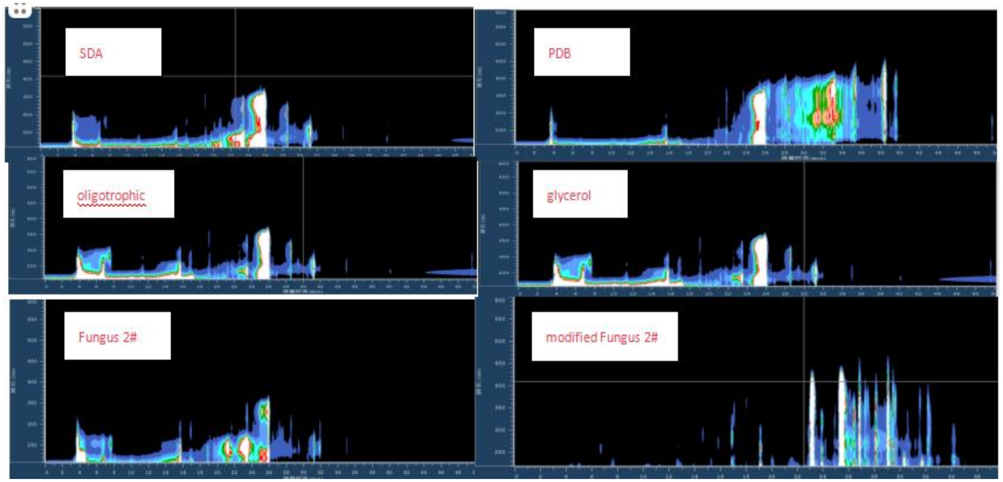

Figure S2. HRESIMS spectrum of compound **1**.

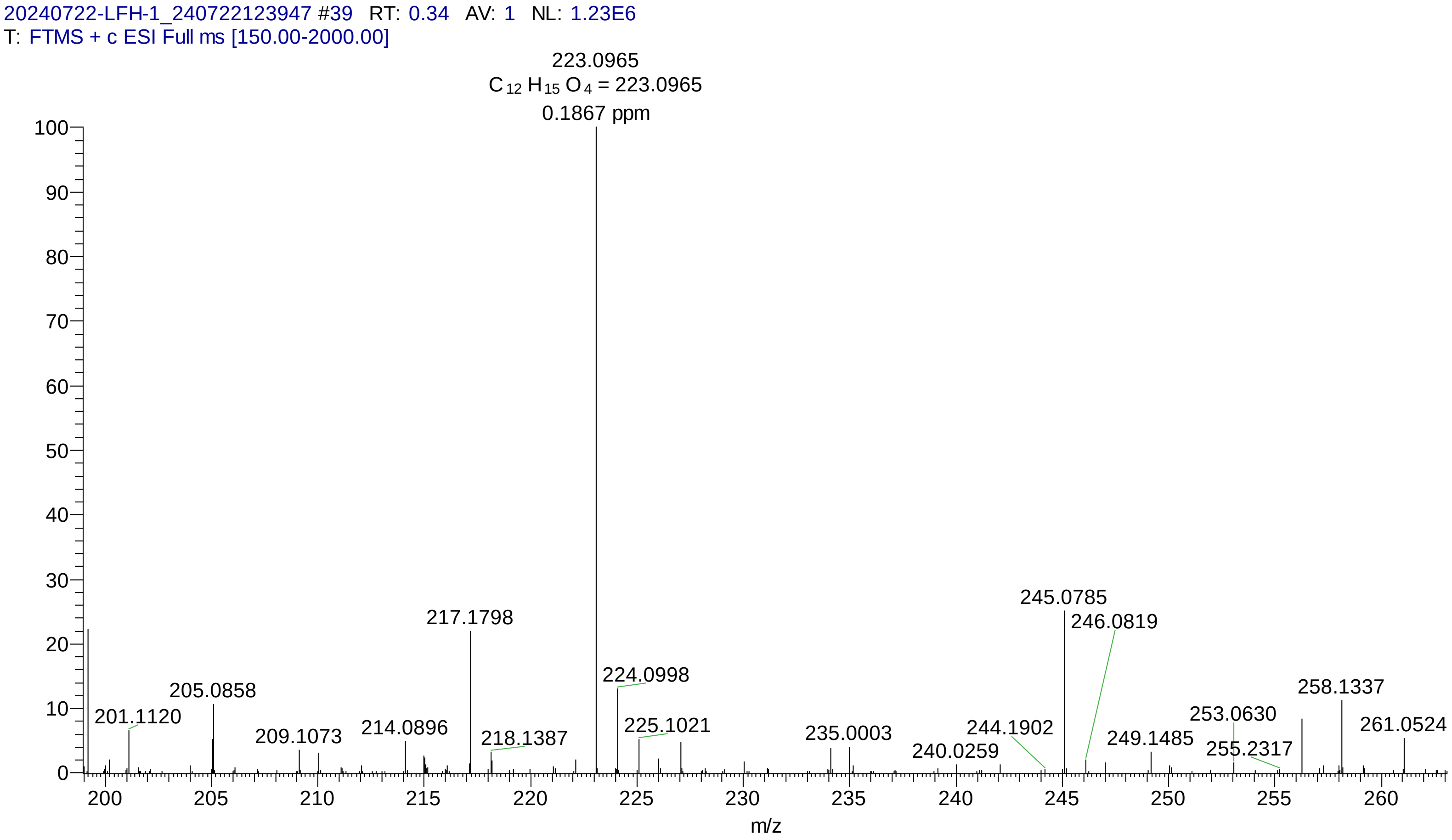

Figure S3. ^{1}H NMR (400 MHz, MeOD-d_4) spectrum of compound **1**.

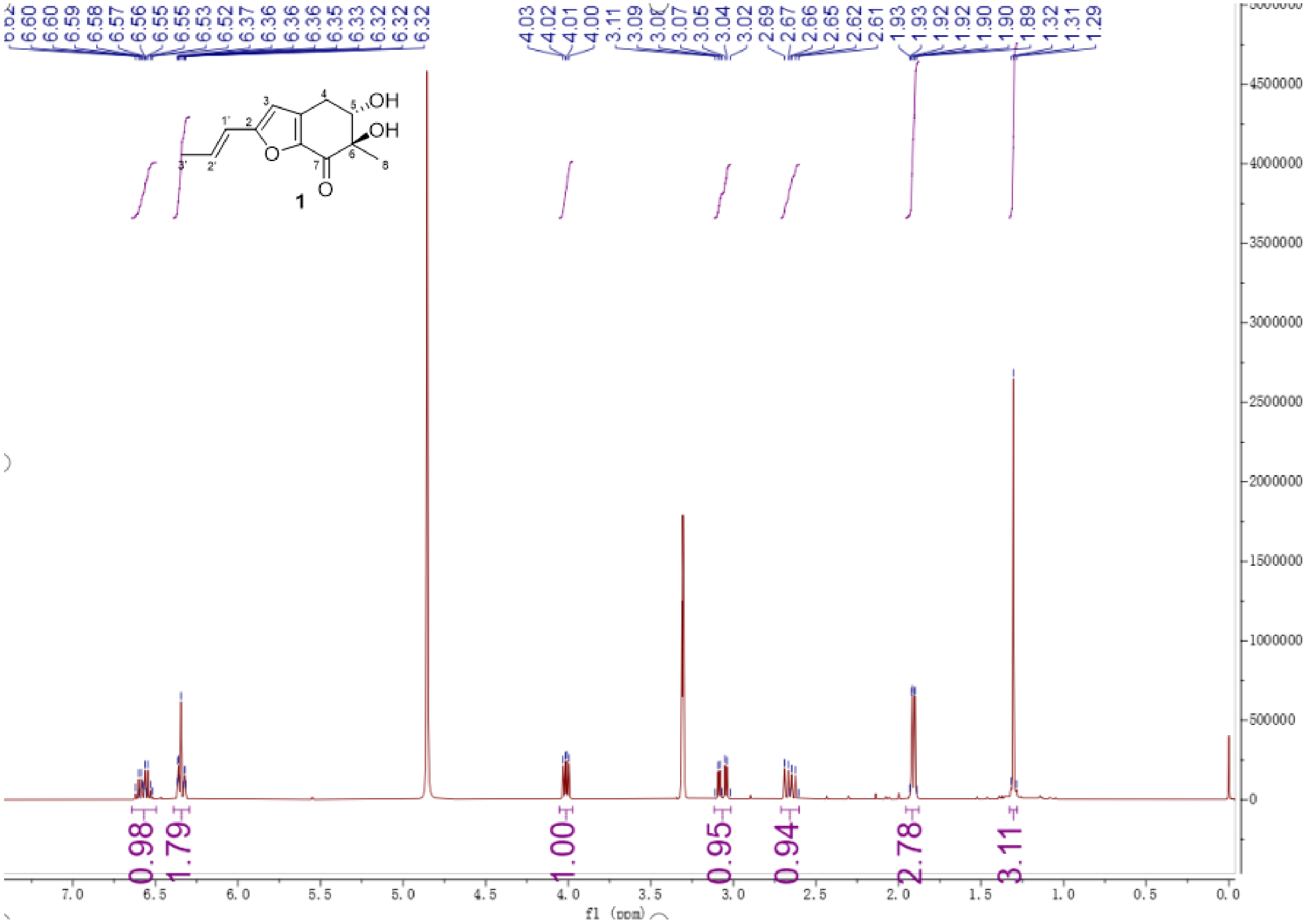

Figure S4. ^{13}C NMR (100 MHz, MeOD-d_4) spectra of compound **1**.

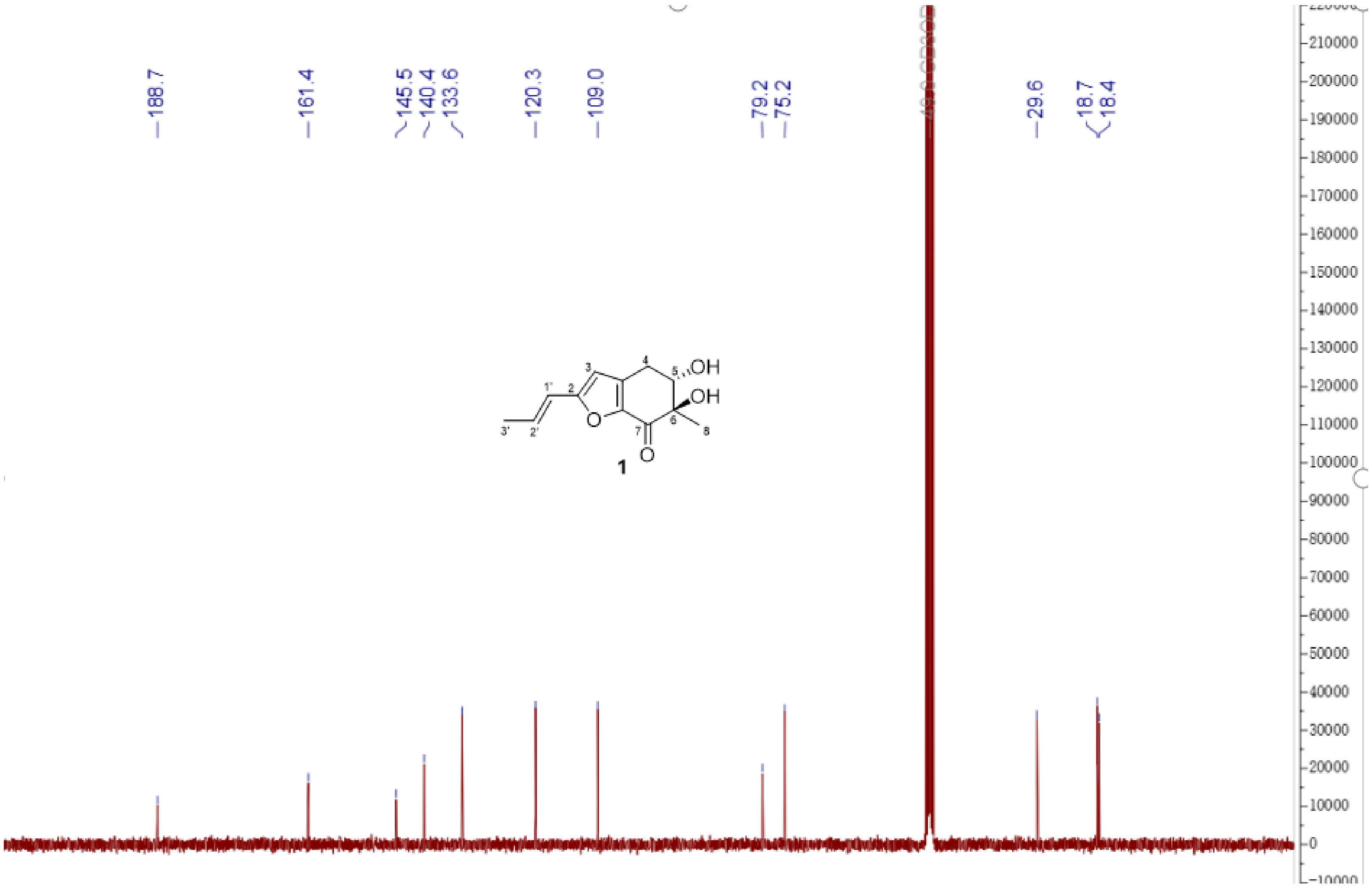

Figure S5. COSY spectrum of compound **1**.

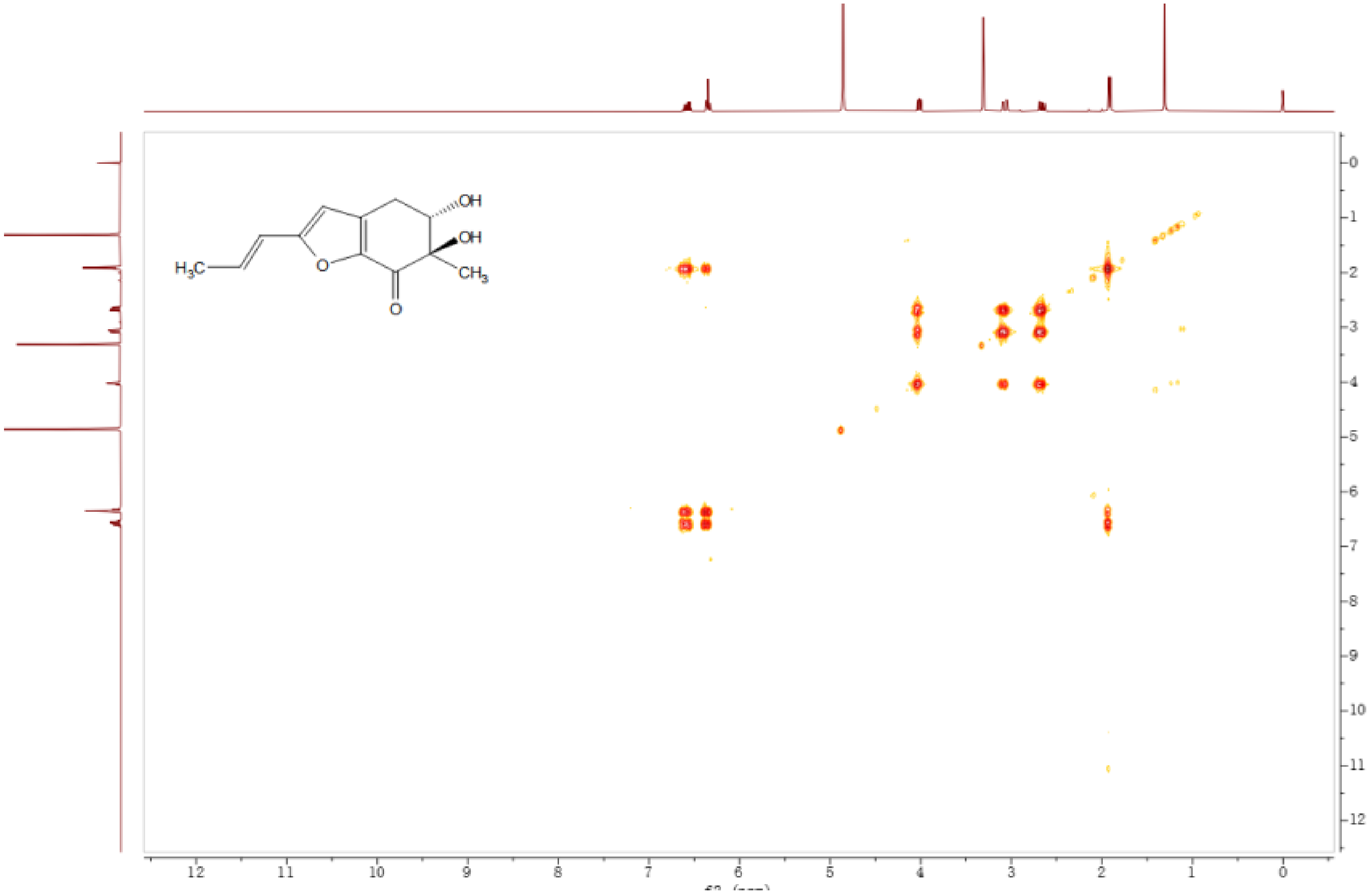

Figure S6. HSQC spectrum of compound **1**.

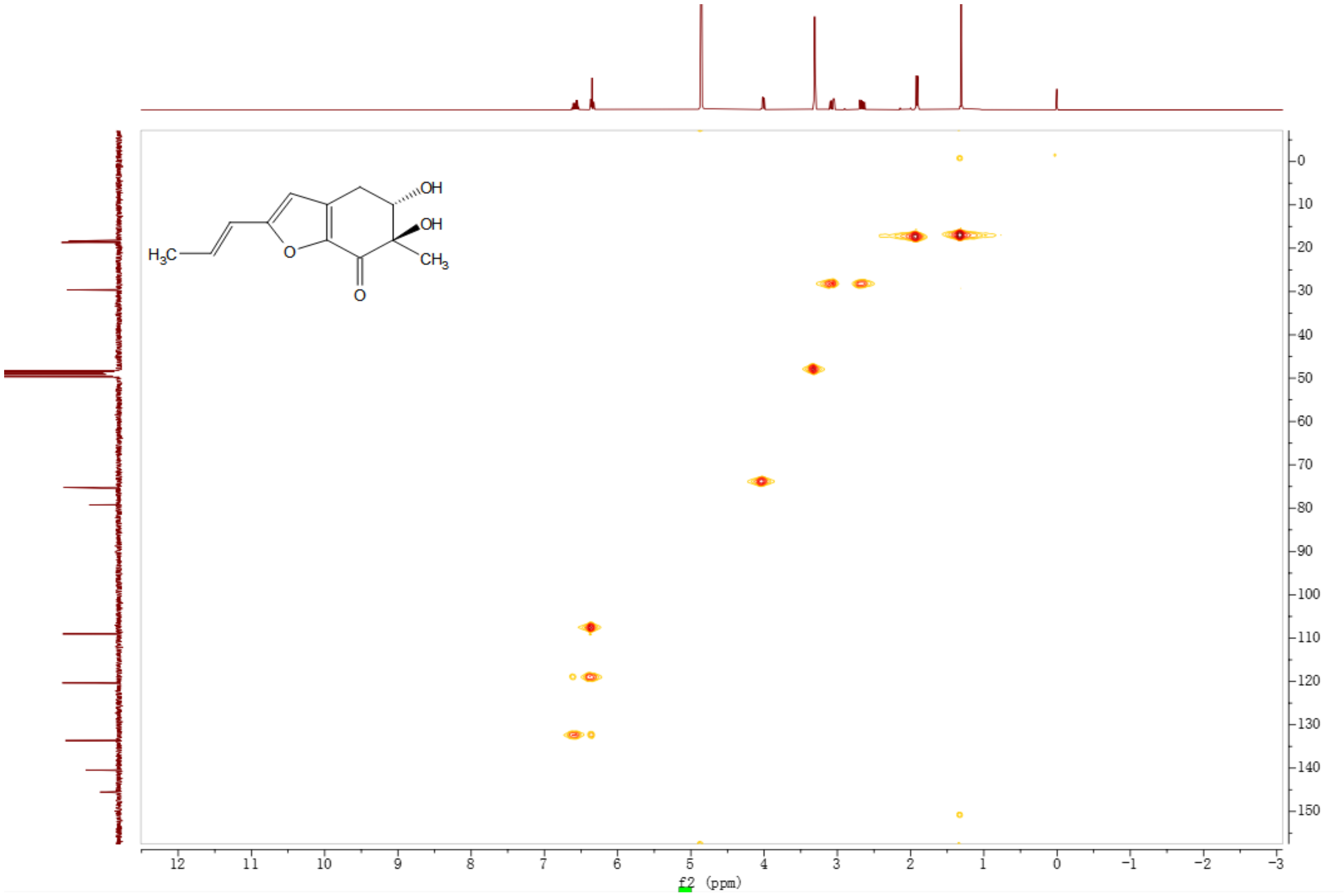

Figure S7. HMBC spectrum of compound **1**.

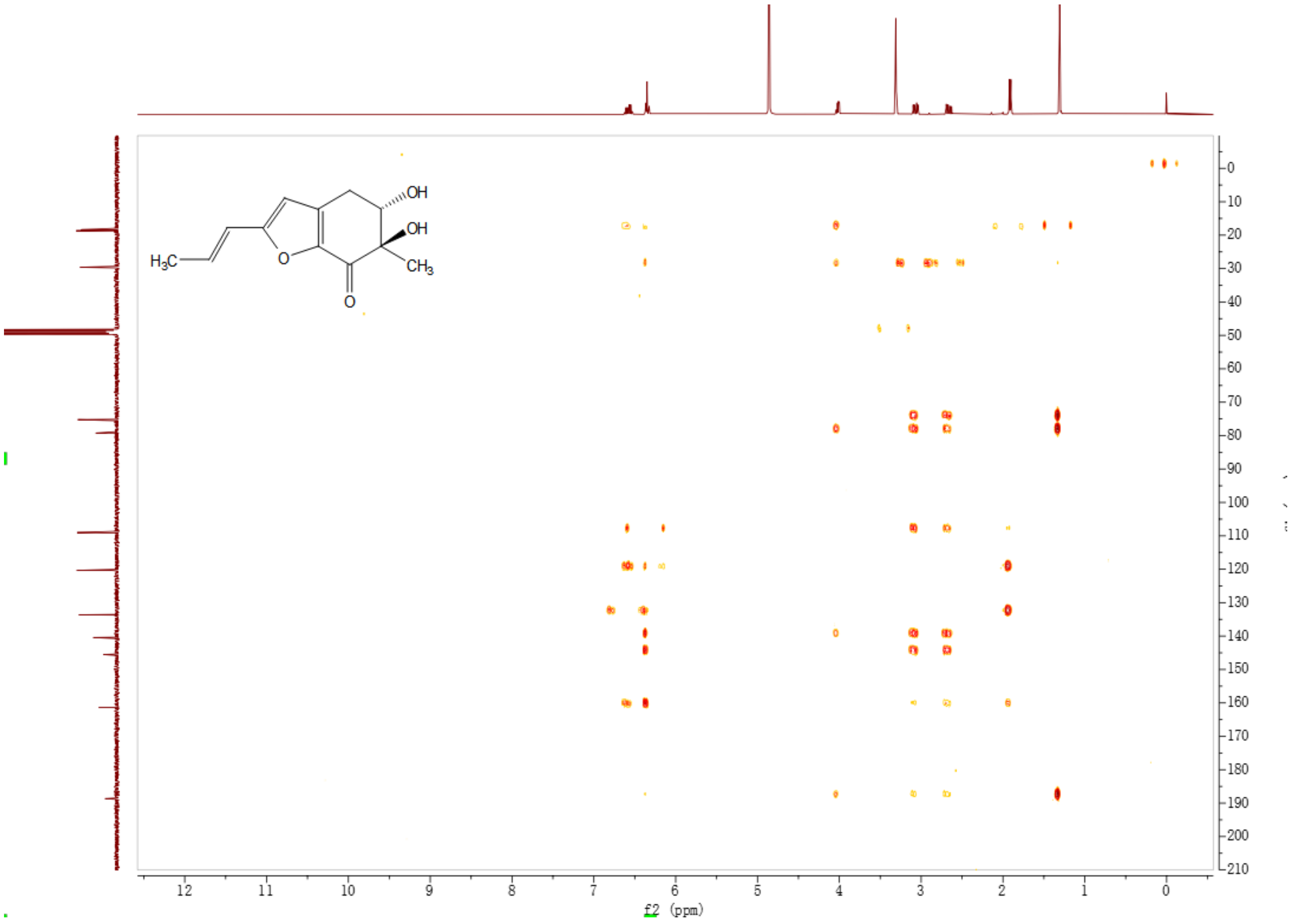

Figure S8. NOE spectrum of **1**.

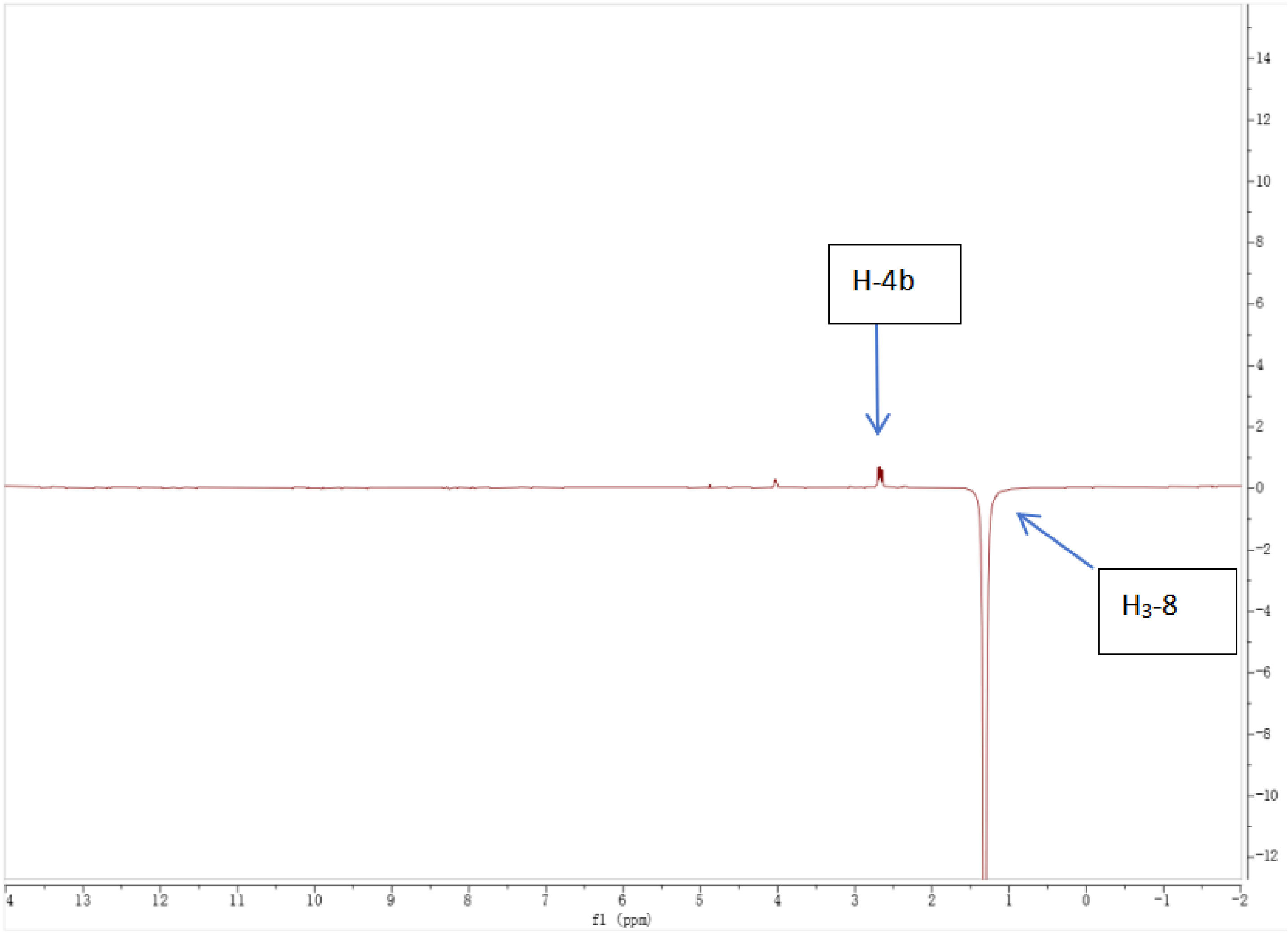

Figure S9. The corresponding structure when DP4+ calculating the relative configuration of compound **1**;

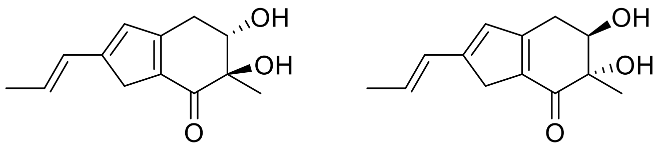

1a

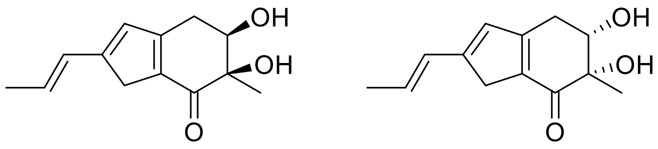

1b

Figure S10. UV spectrum of compound **1**

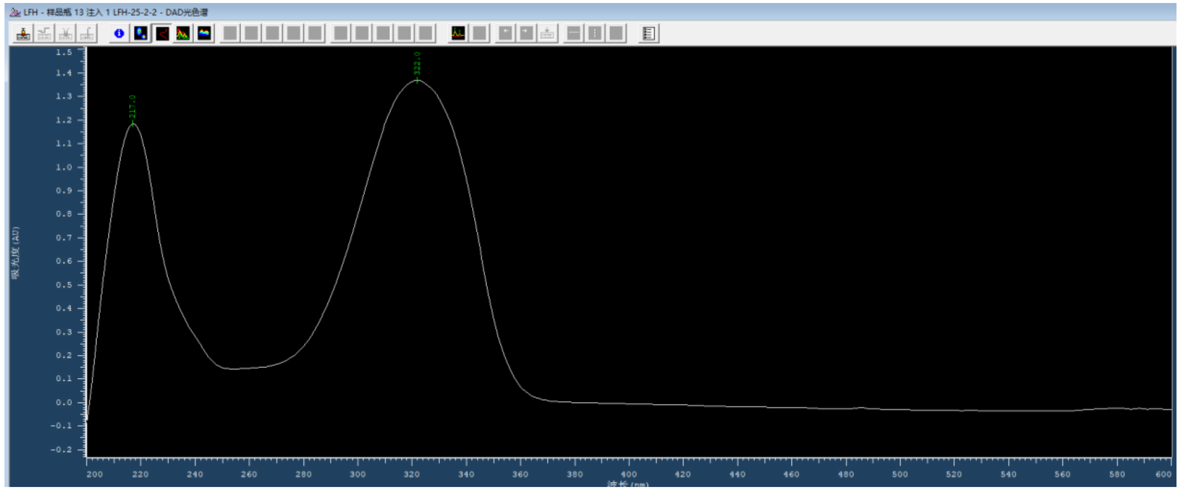

Figure S11. IR spectrum of compound **1**

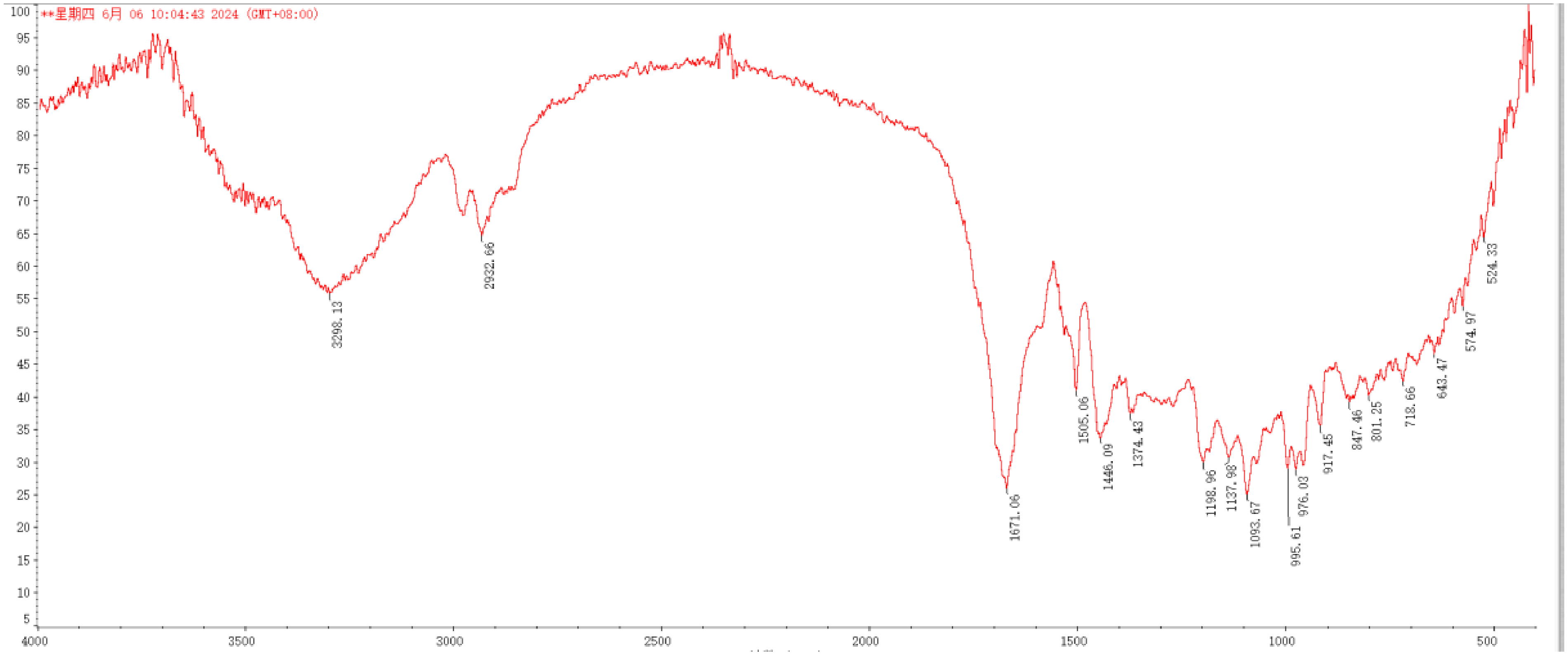

Figure S12. HRESIMS spectrum of compound **2**.

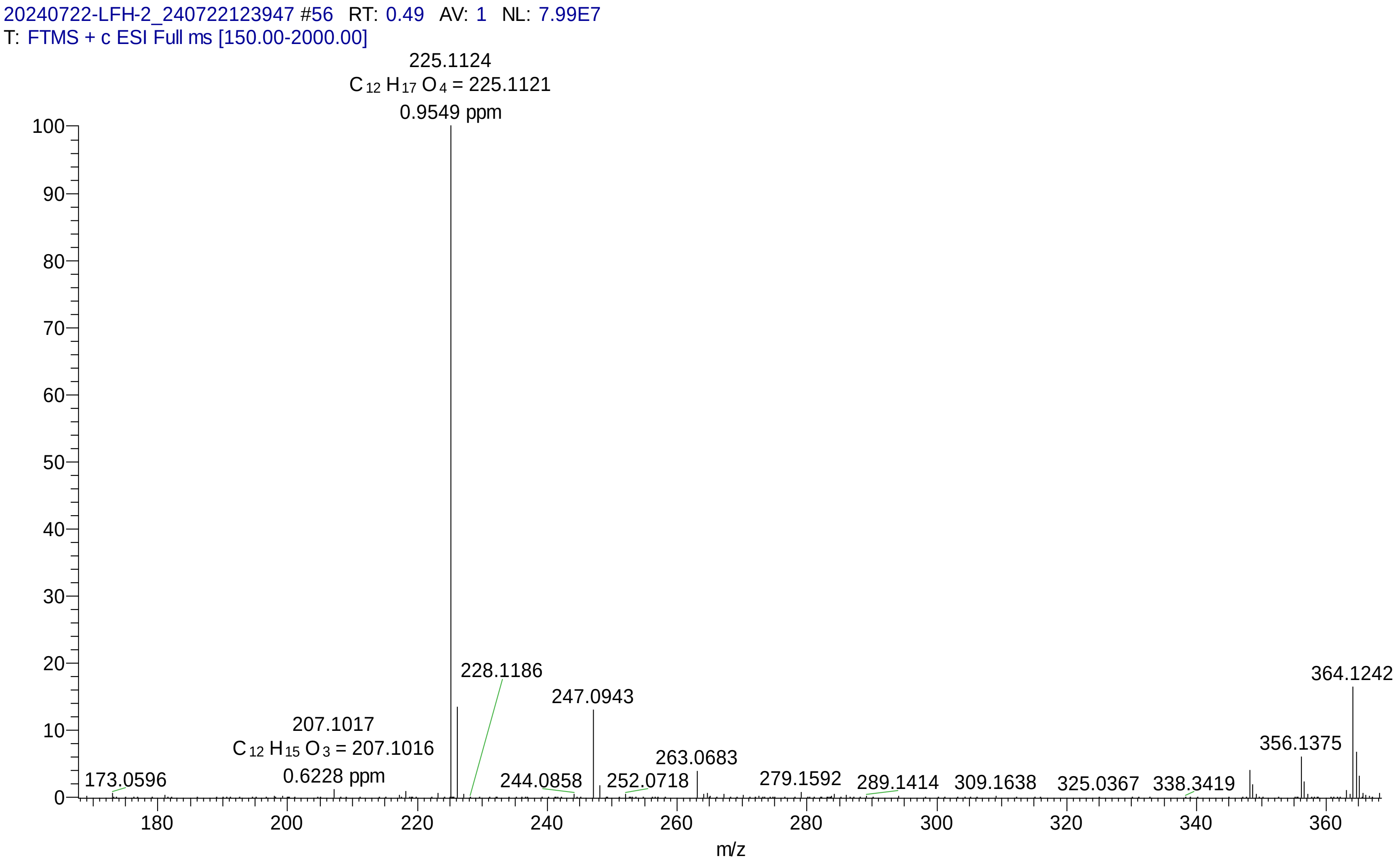

Figure S13. ¹H NMR (4000 MHz, DMSO-*d6*) spectrum of compound **2**.

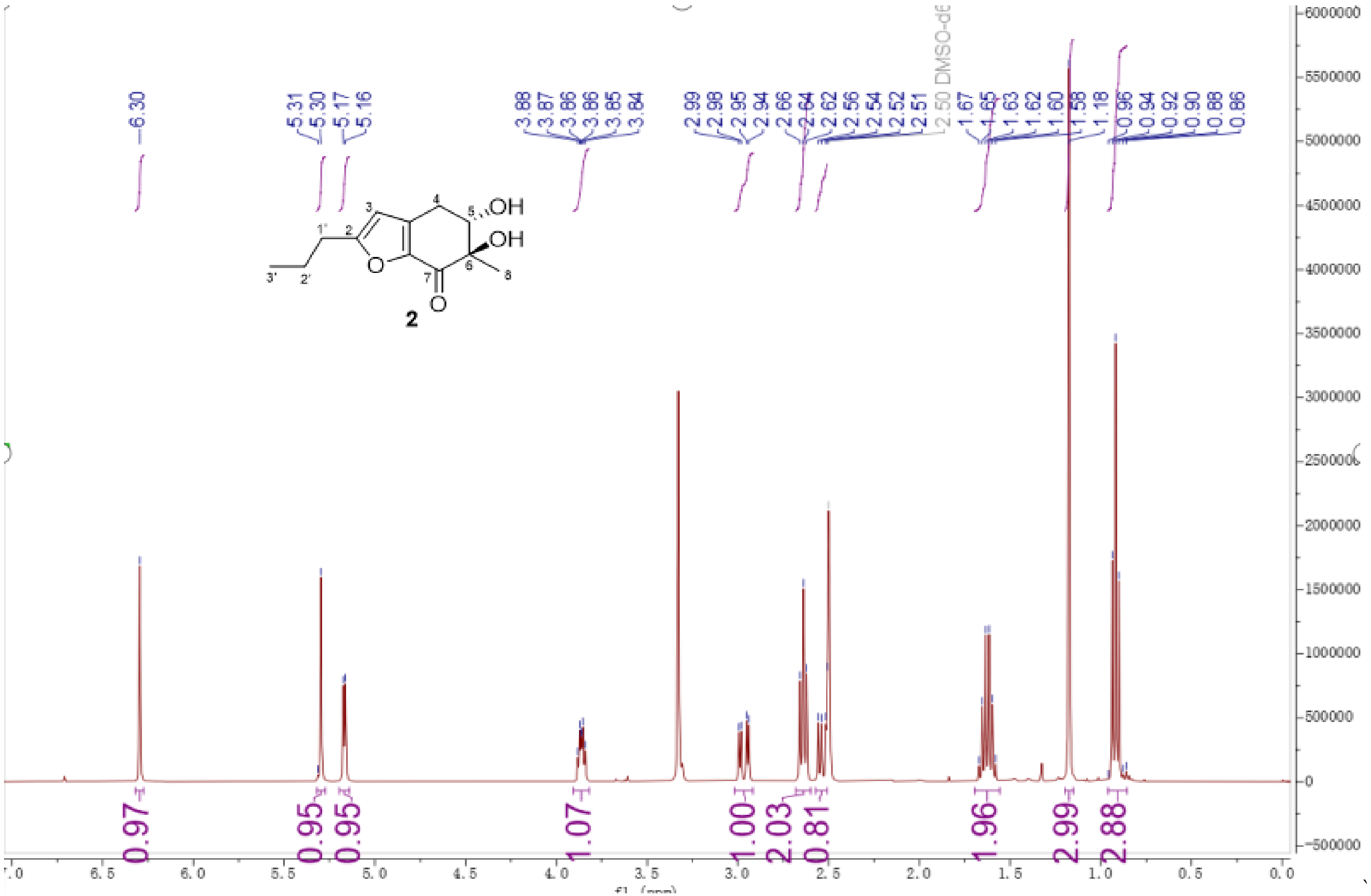

Figure S14. ^{13}C NMR (125 MHz, DMSO-*d*6) of compound **2**.

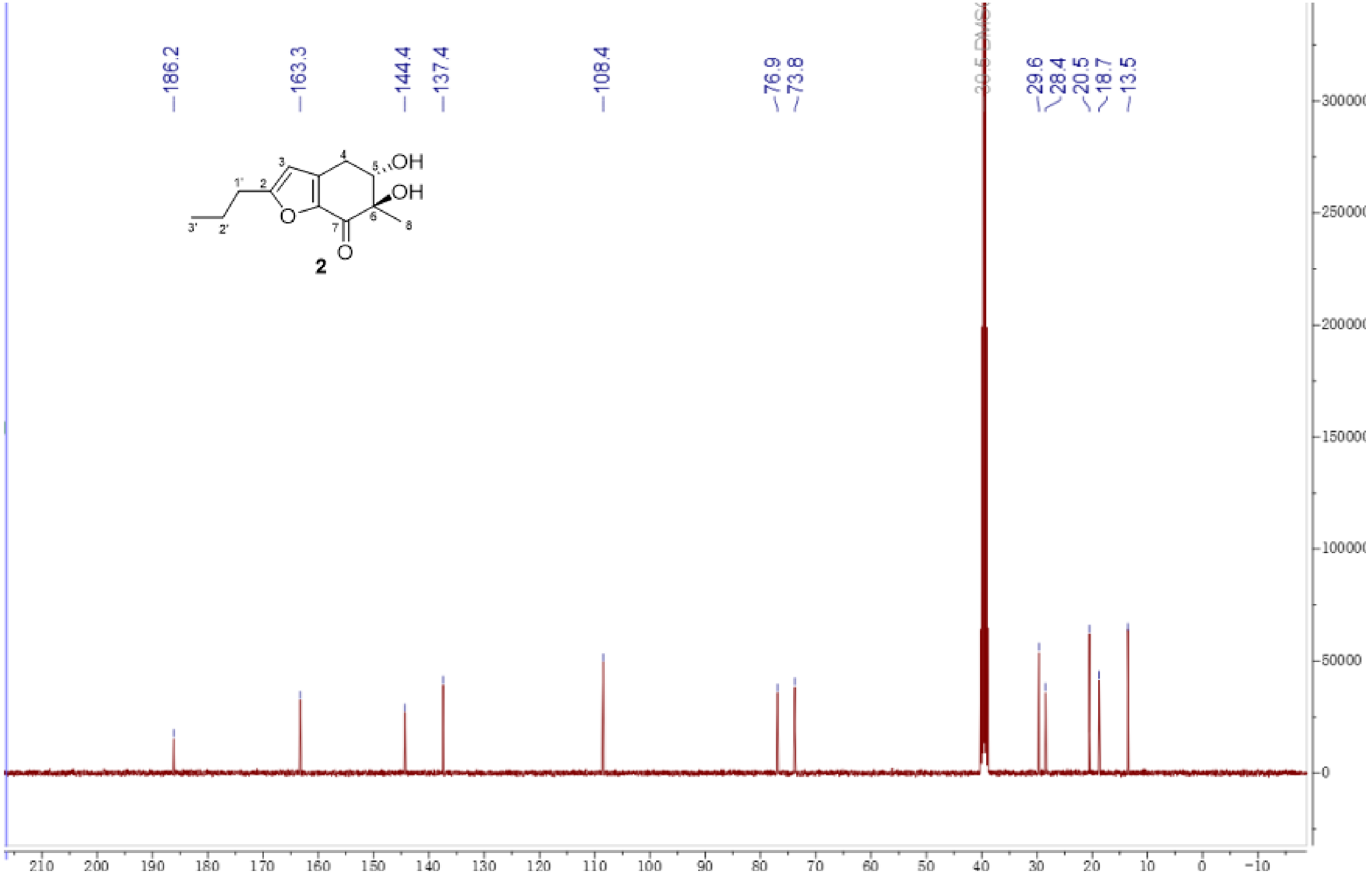

Figure S15. COSY spectrum of compound **2**.

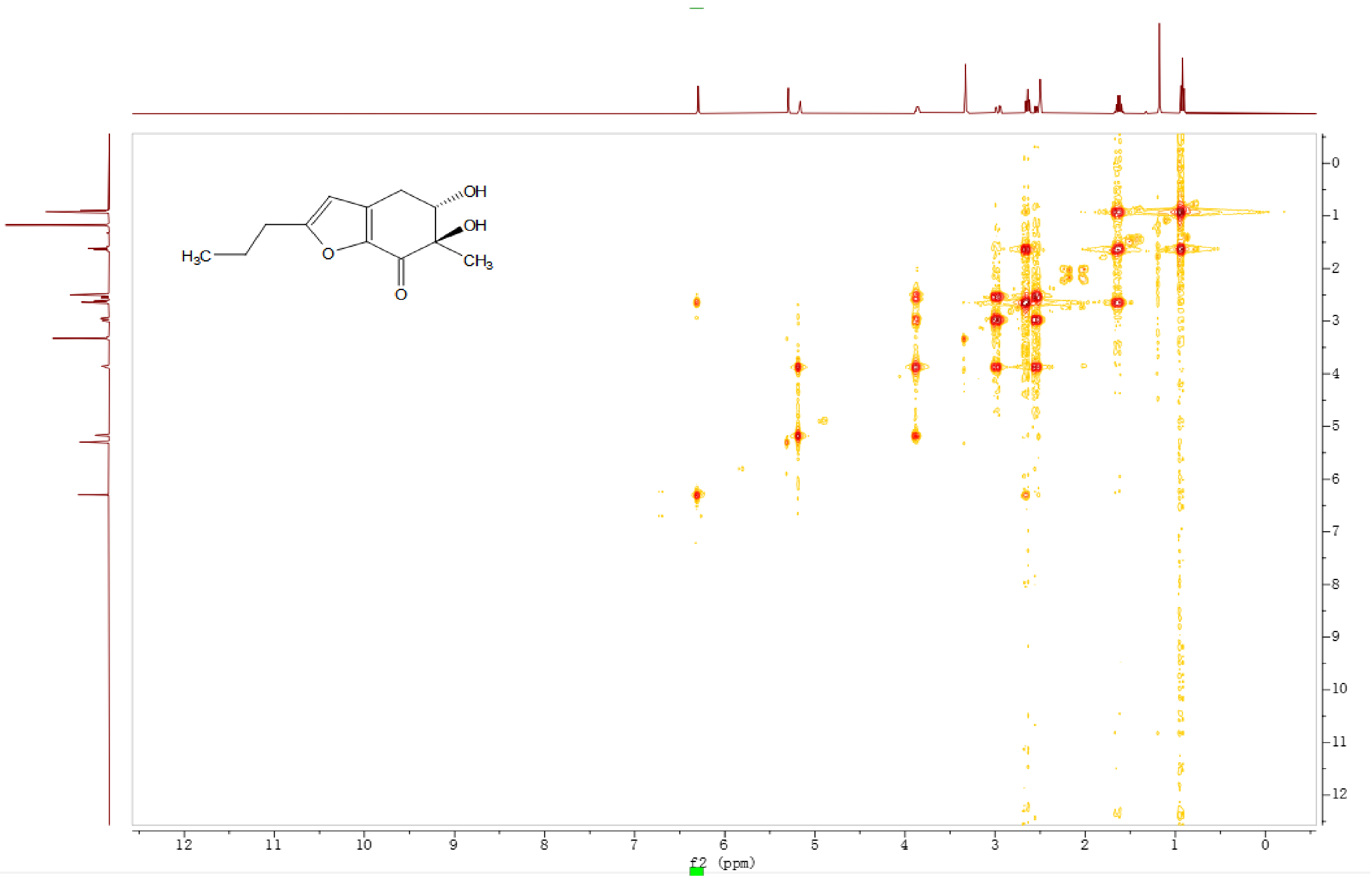

Figure S16. HSQC spectrum of compound **2**.

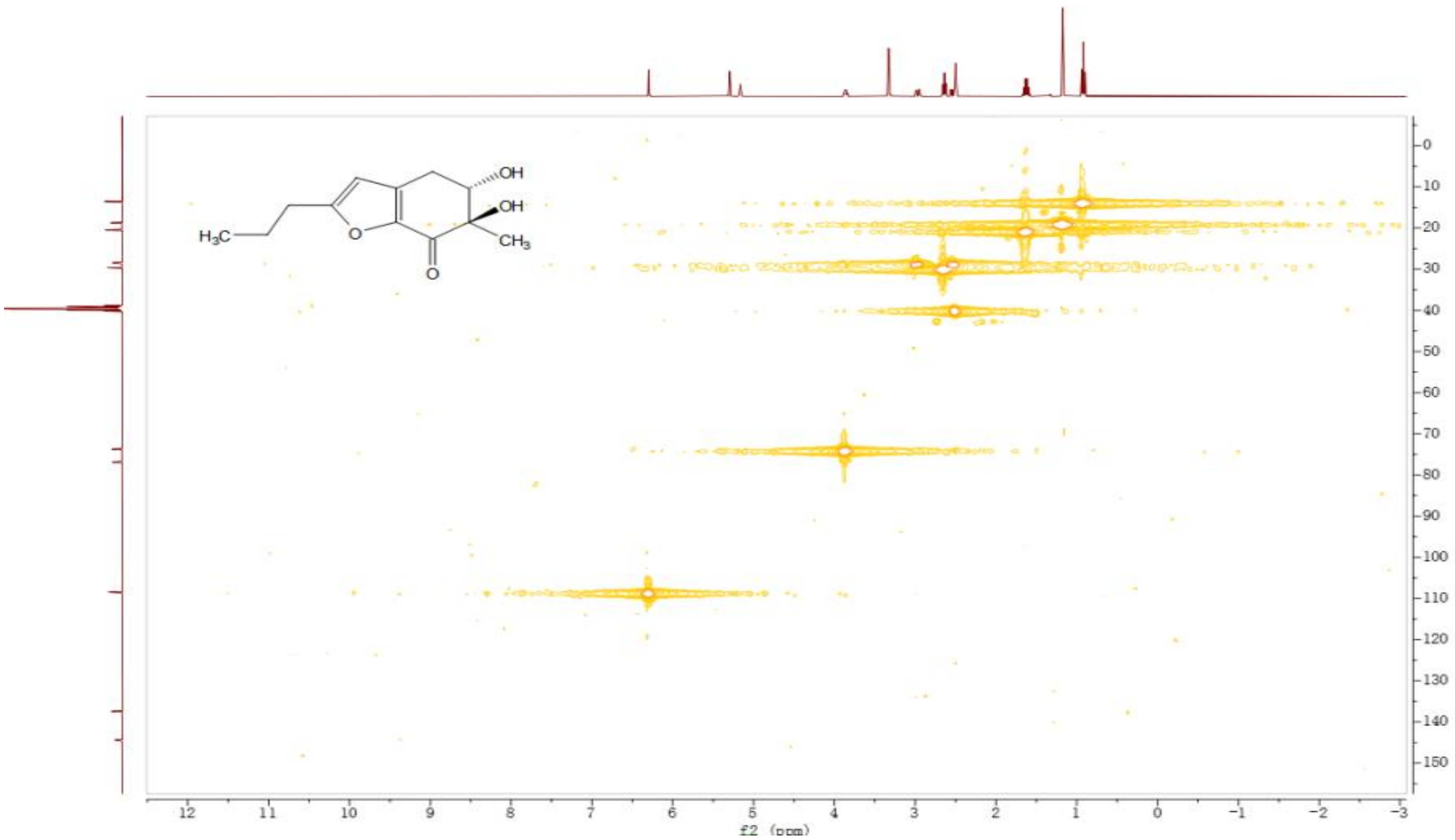

Figure S17. HMBC spectrum of compound **2**.

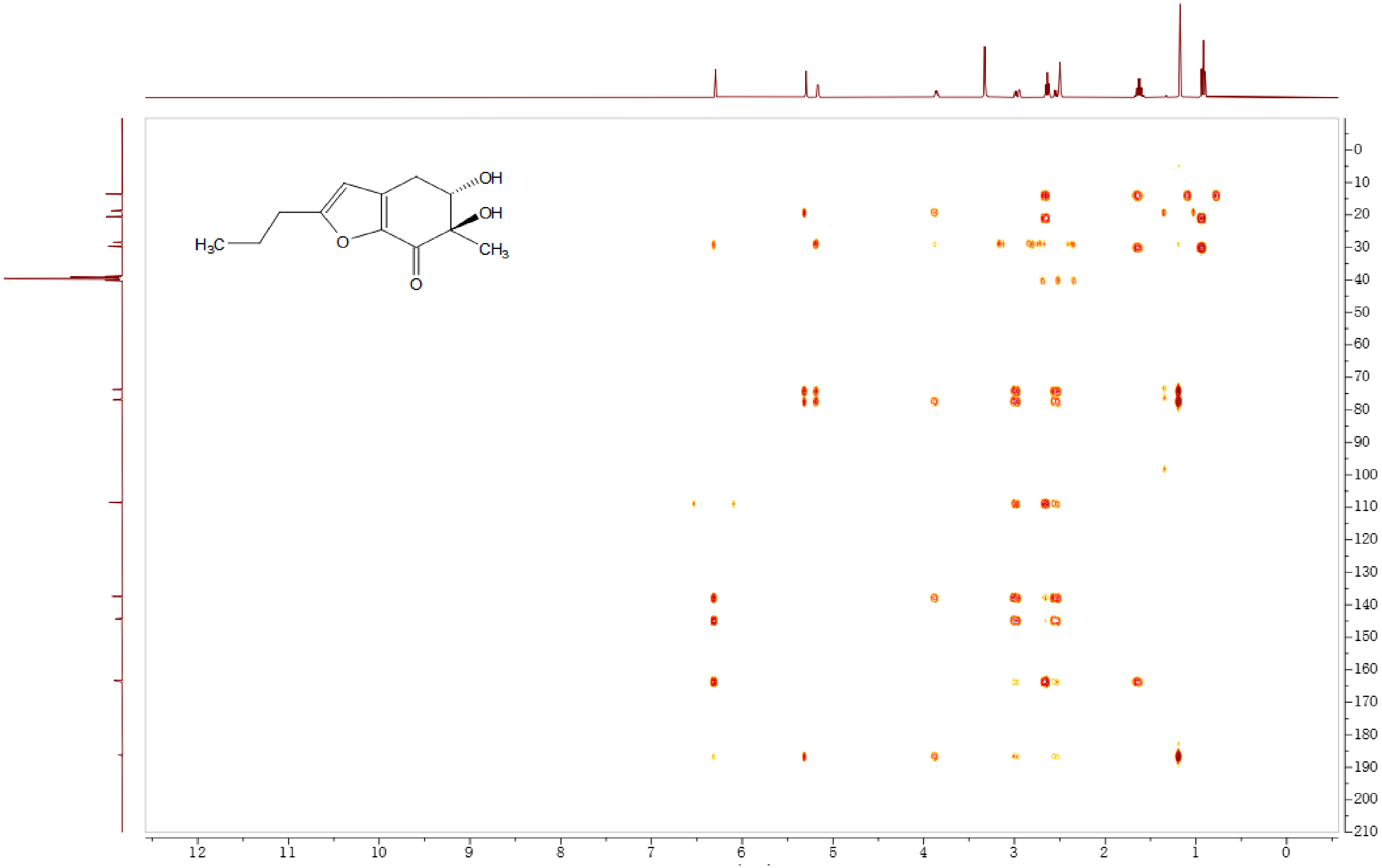

Figure S18. The corresponding structure when DP4+ calculating the relative configuration of compound **2.**

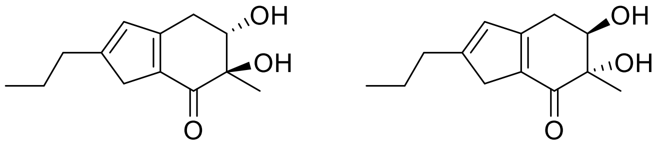

2a

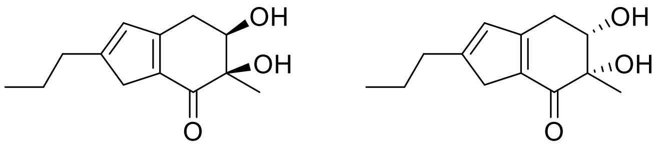

2b

Figure S19. UV spectrum of compound **2**.

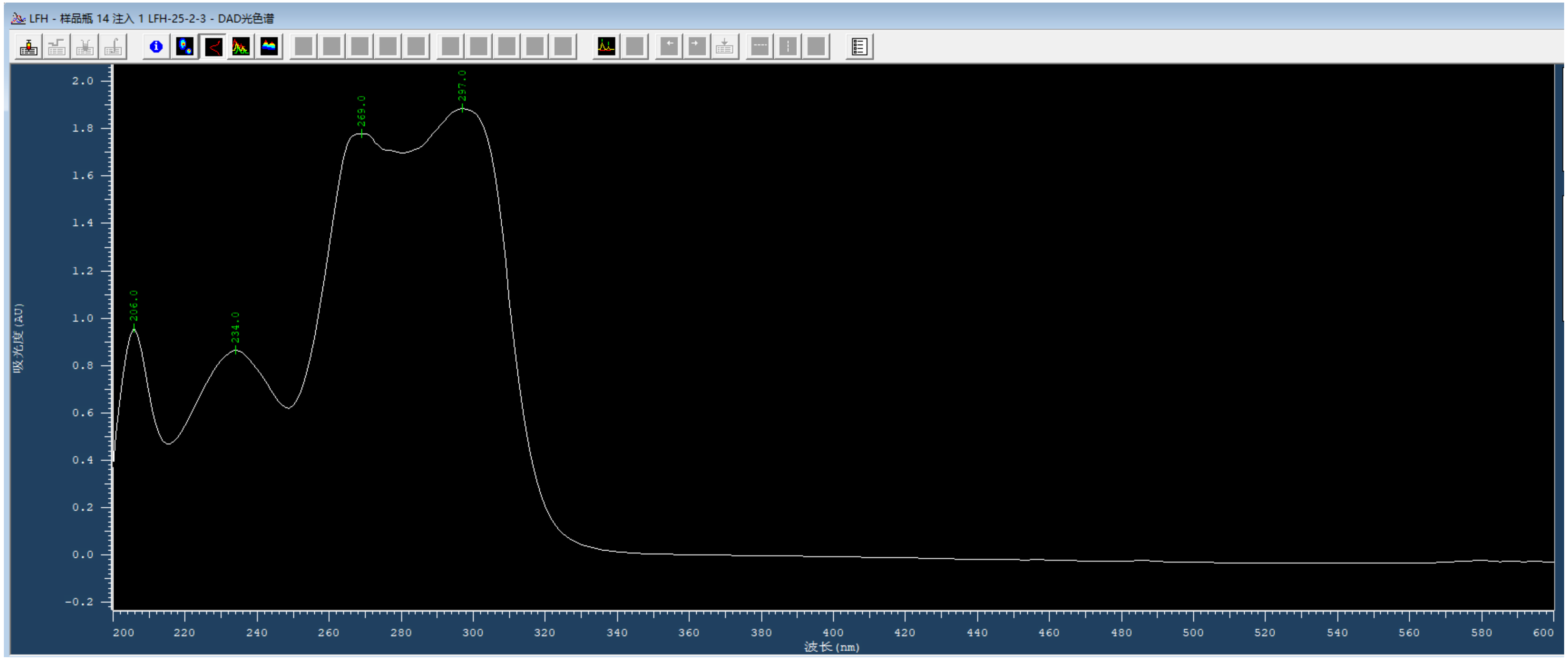

Figure S20. IR spectrum of compound **3**.

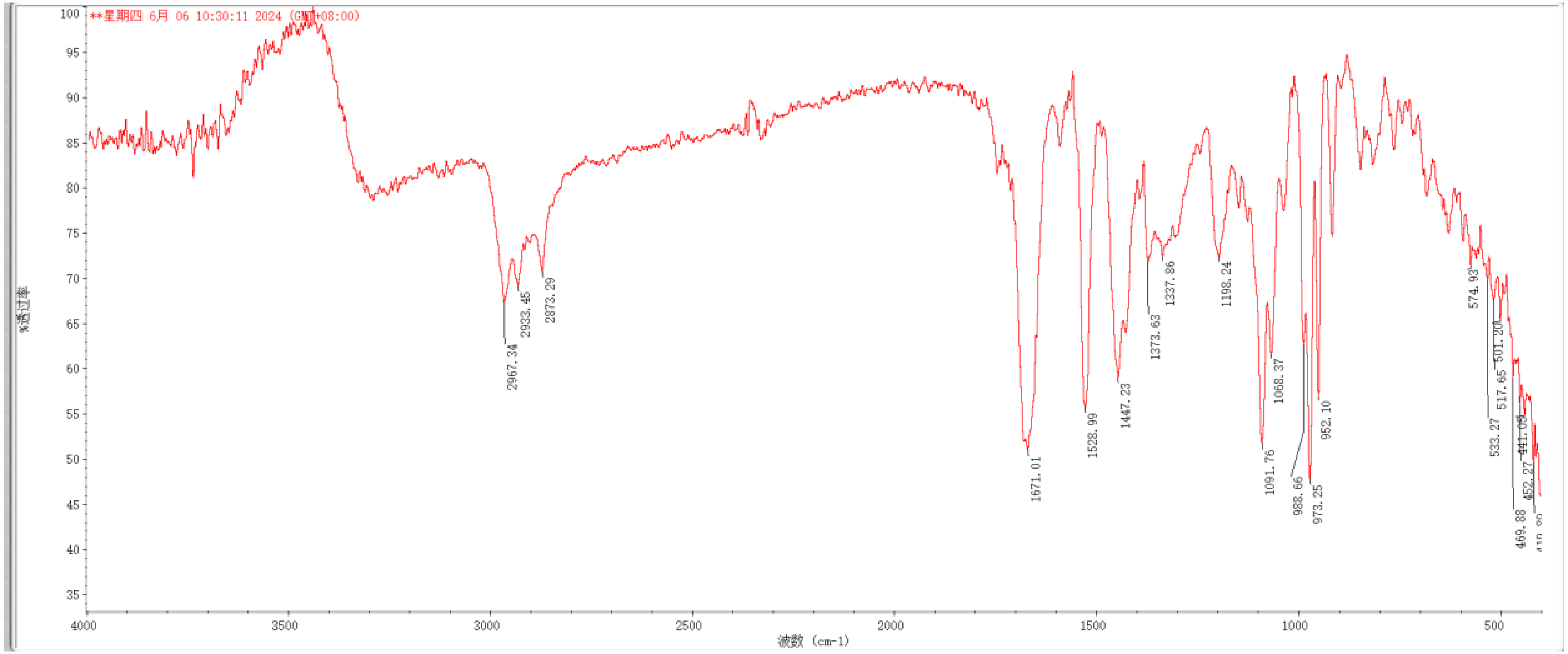

Figure S21. HRESIMS spectrum of compound **3**.

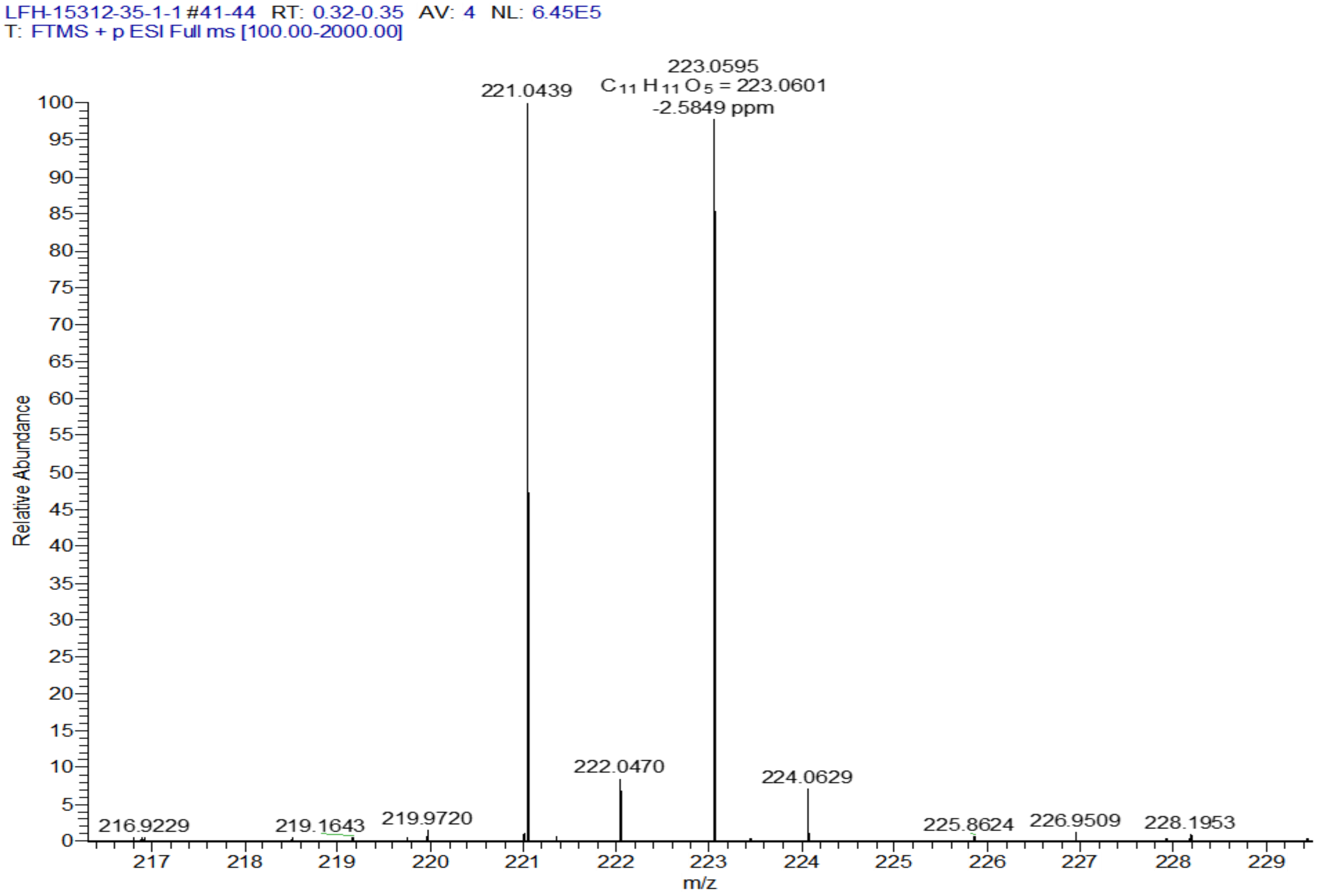

Figure S22. ^{1}H NMR (500 MHz, DMSO-d_6) spectrum of compound **3**.

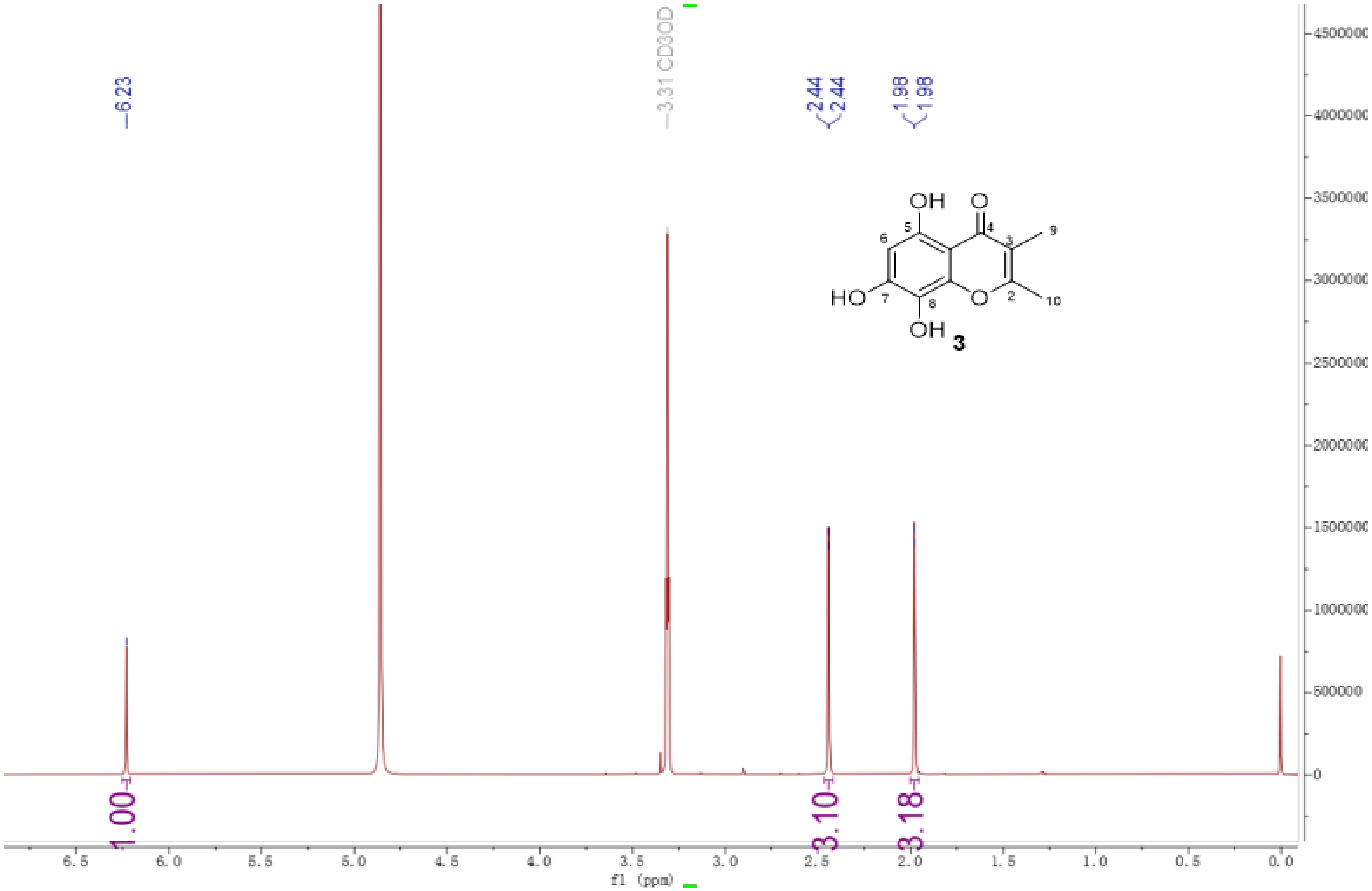

Figure S23 ^{13}C NMR (125 MHz, DMSO-*d6*) spectra of compound **3**.

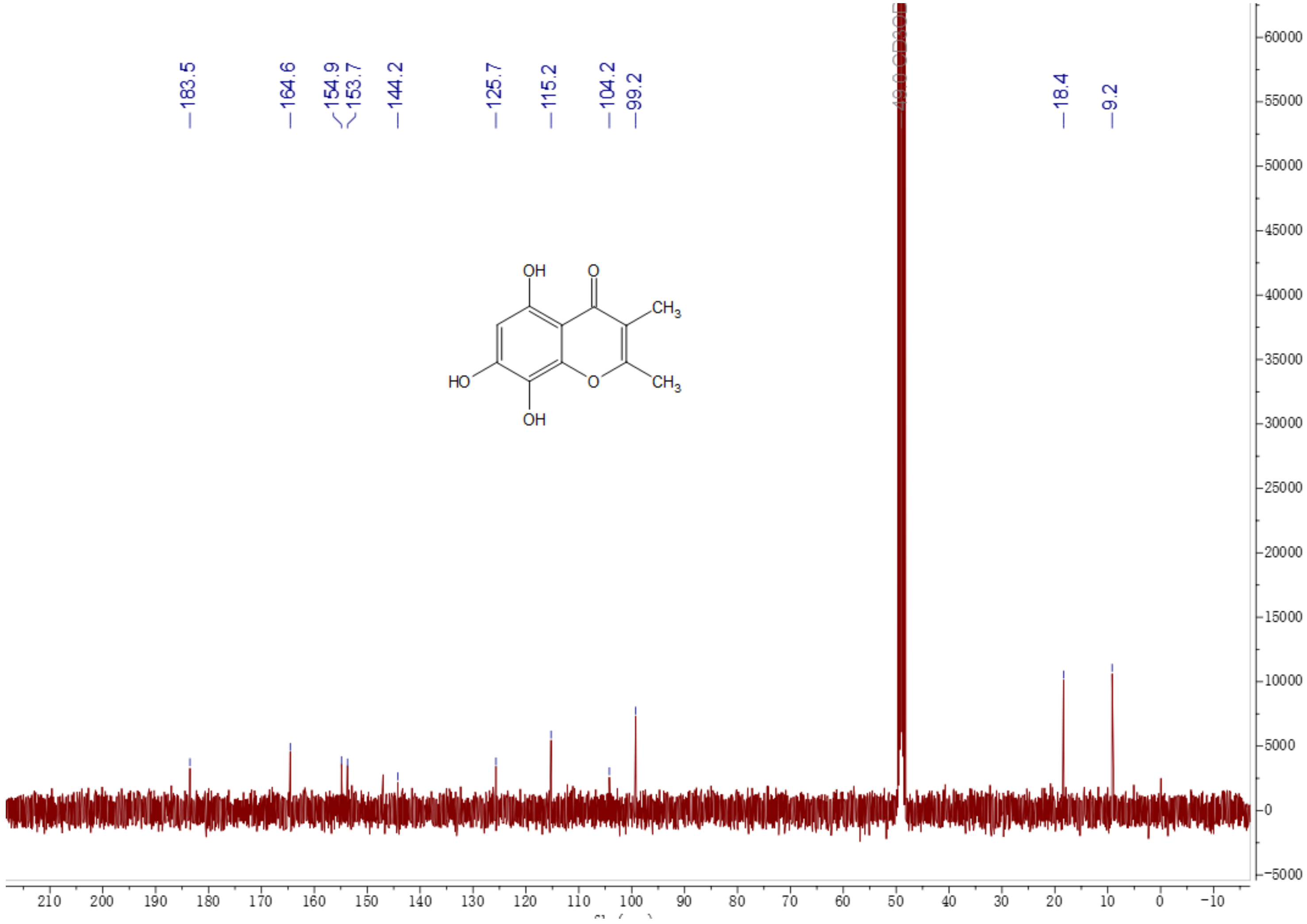

Figure S24. HSQC spectrum of compound **3**.

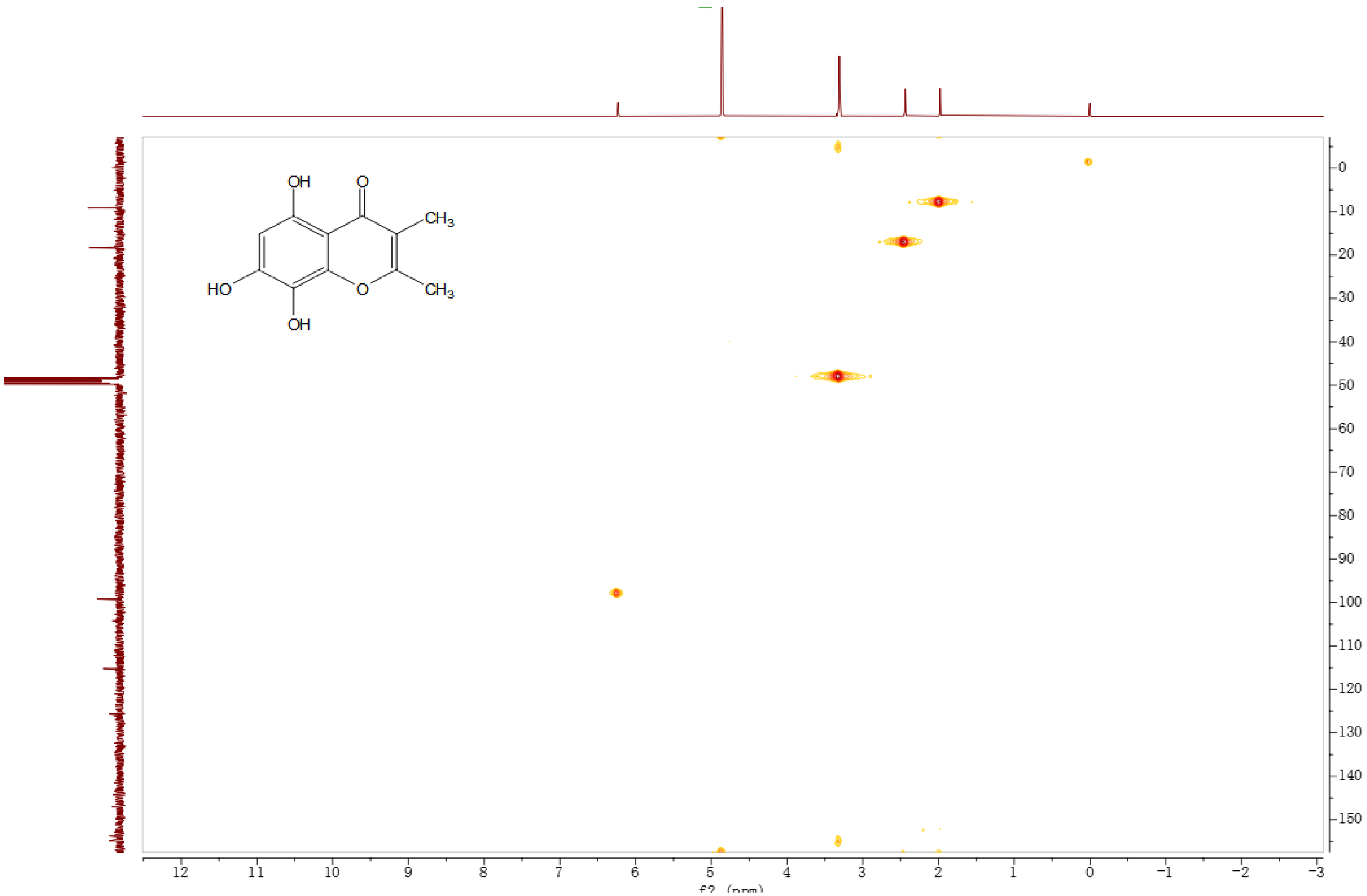

Figure S25. HMBC spectrum of compound **3**.

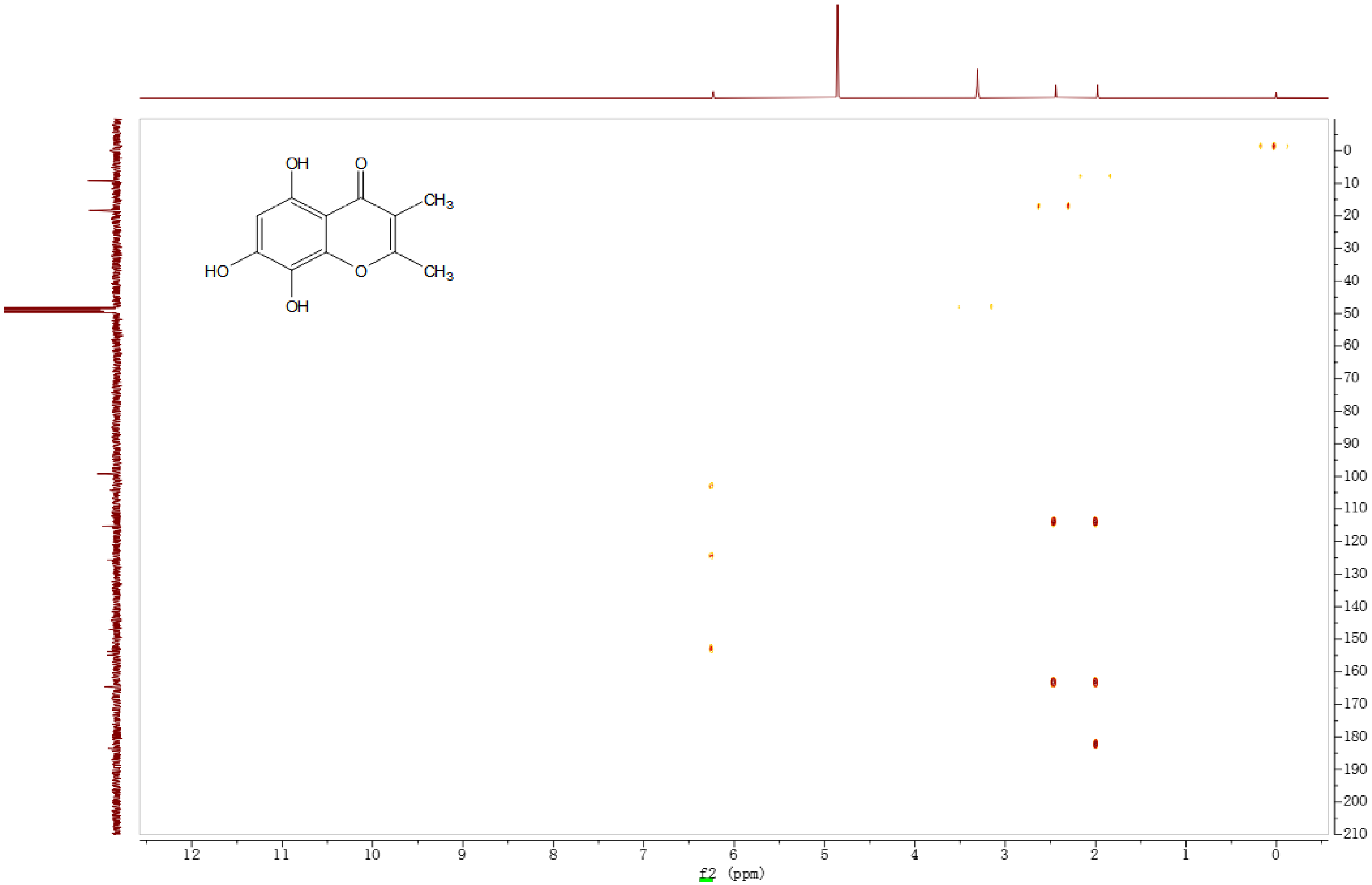

Figure S26. UV spectrum of compound **3**.

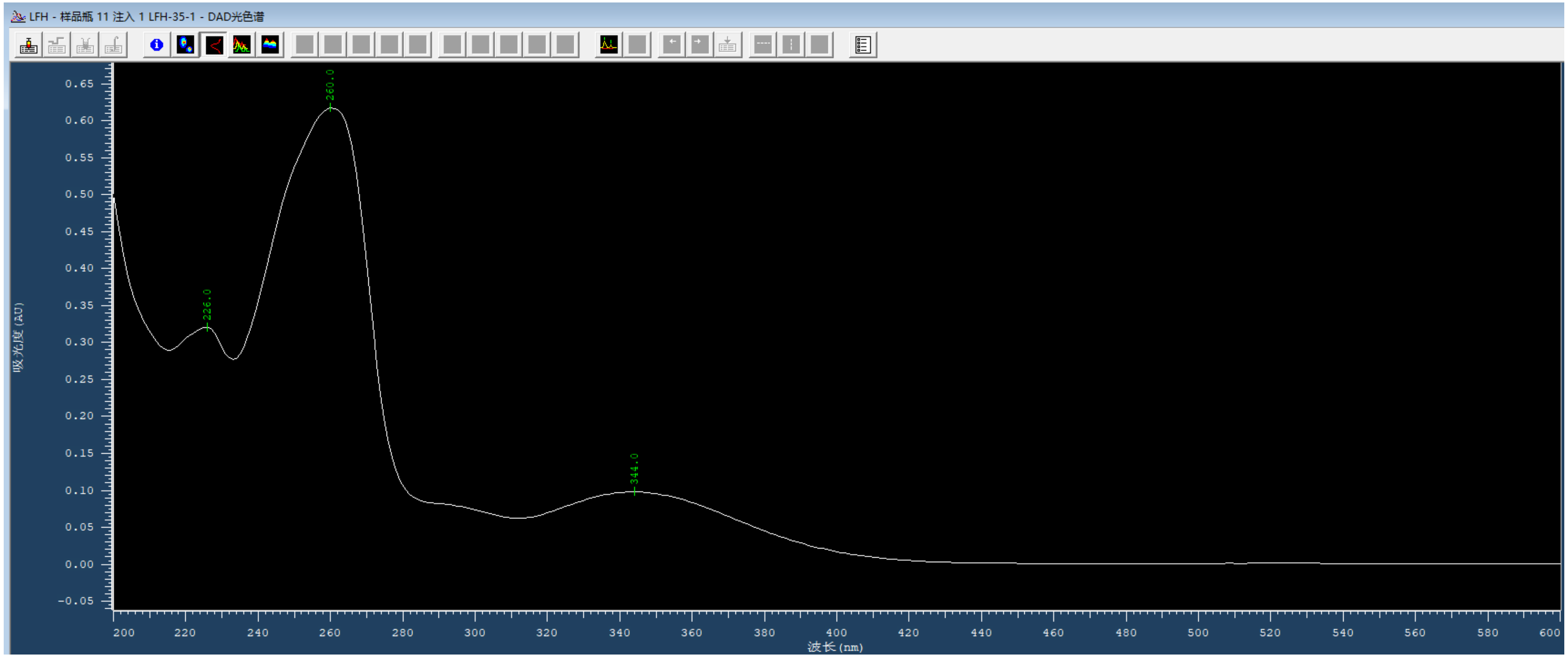

Figure S27. IR spectrum of compound **3**.

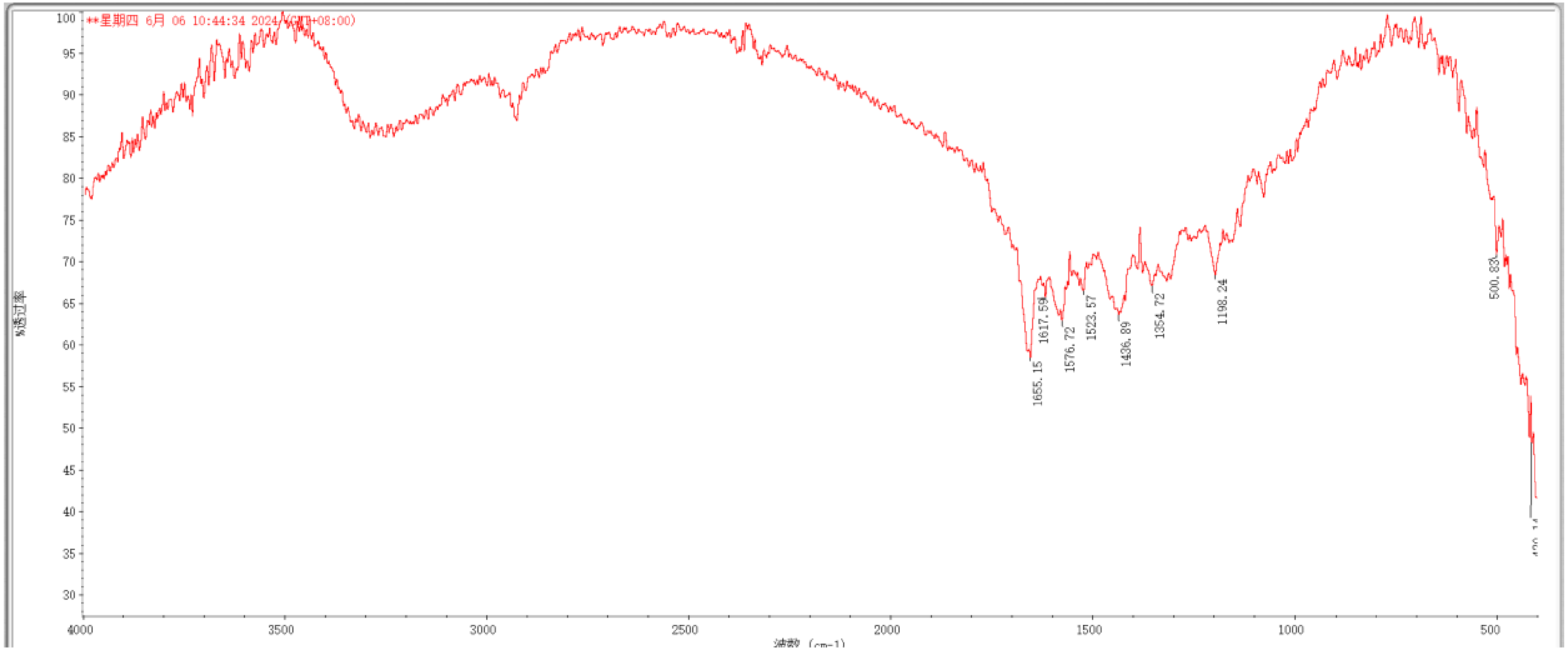

Figure S28. HRESIMS spectrum of compound **4**.

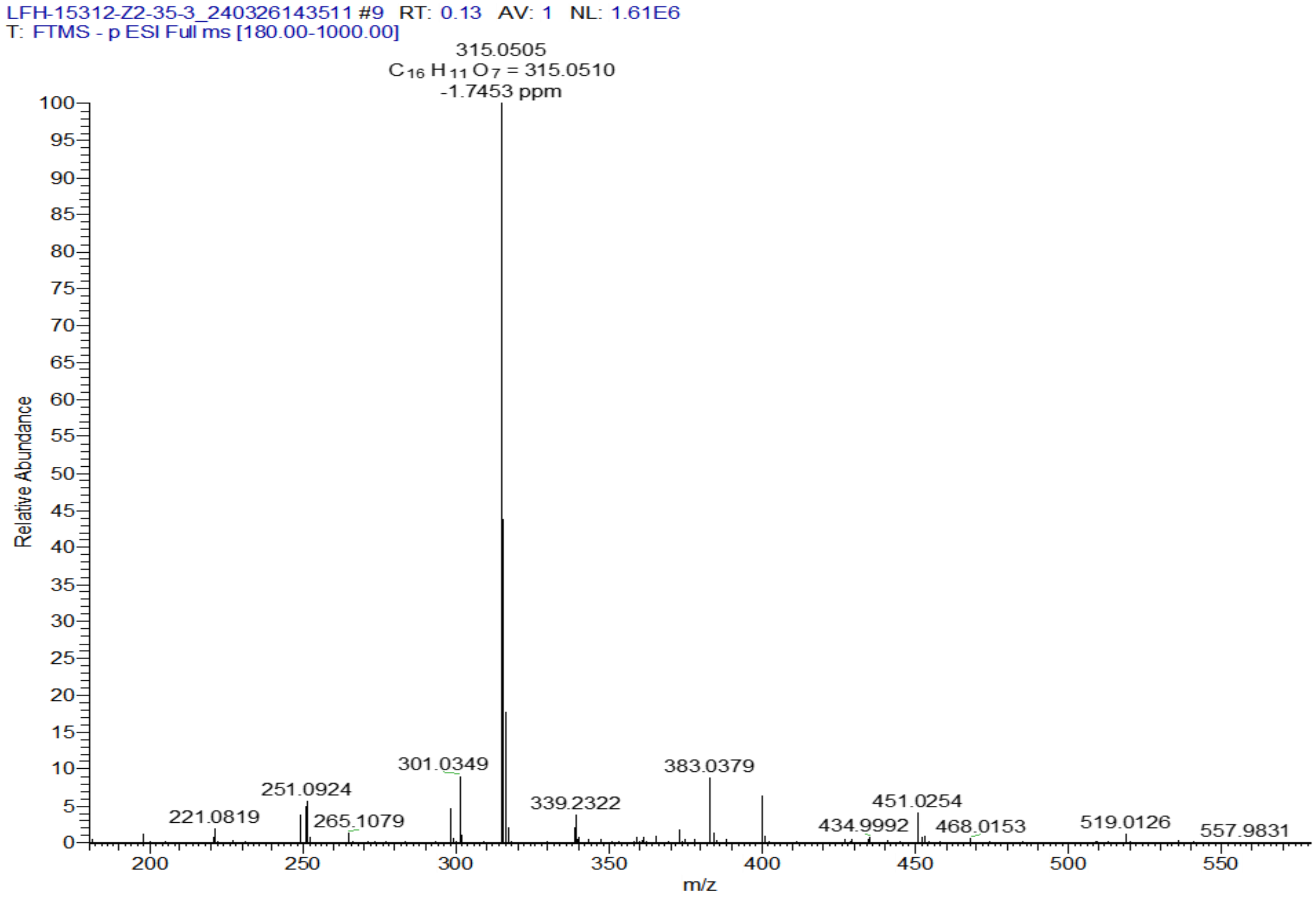

Figure S29. ^{1}H NMR (400 MHz, DMSO-d_6) spectrum of compound **4**.

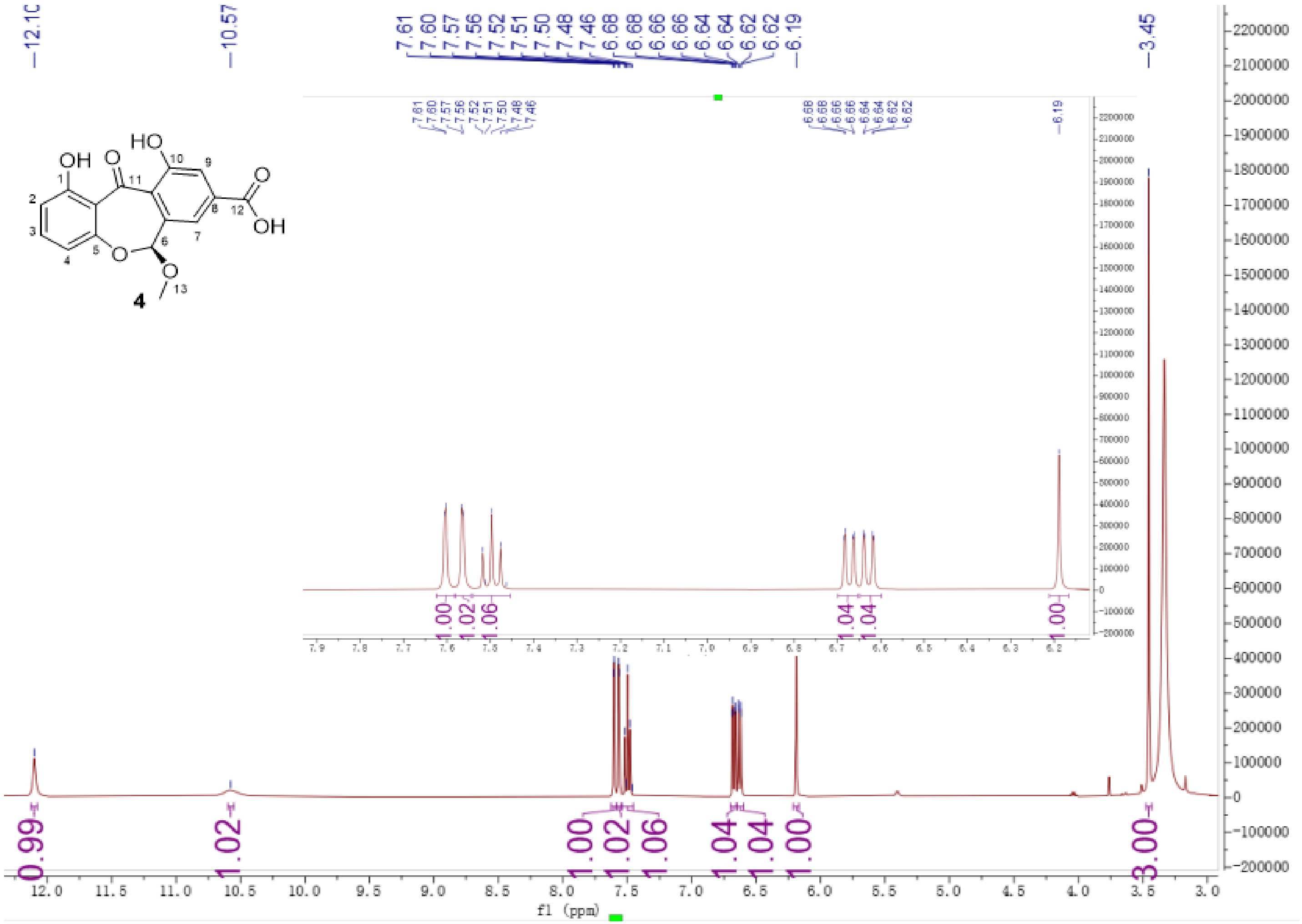

Figure S30. ^{13}C NMR (125 MHz, DMSO-d_6) of compound **4**.

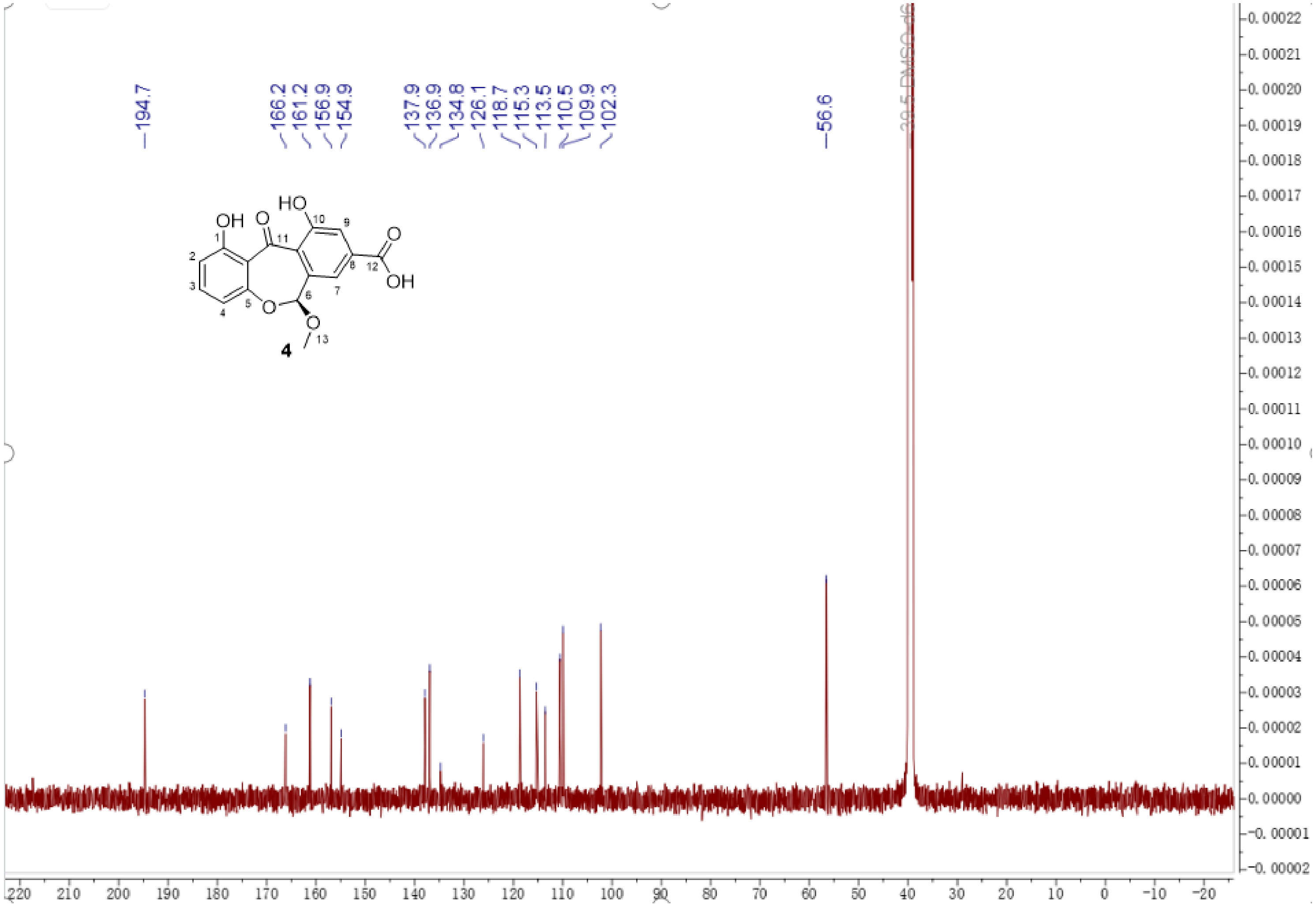

Figure S31. COSY spectrum of compound **4**.

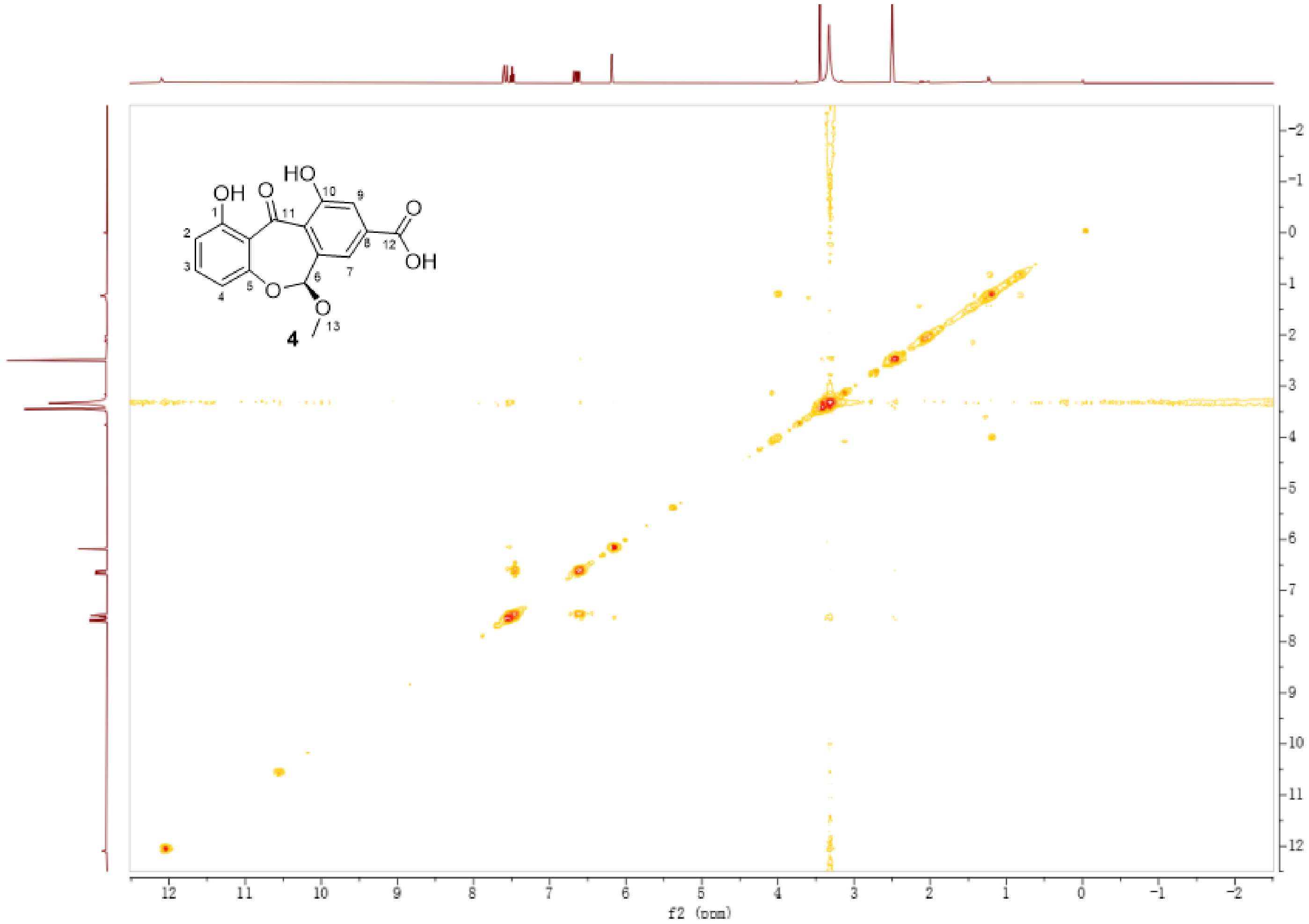

Figure S32. HSQC spectrum of compound **4**.

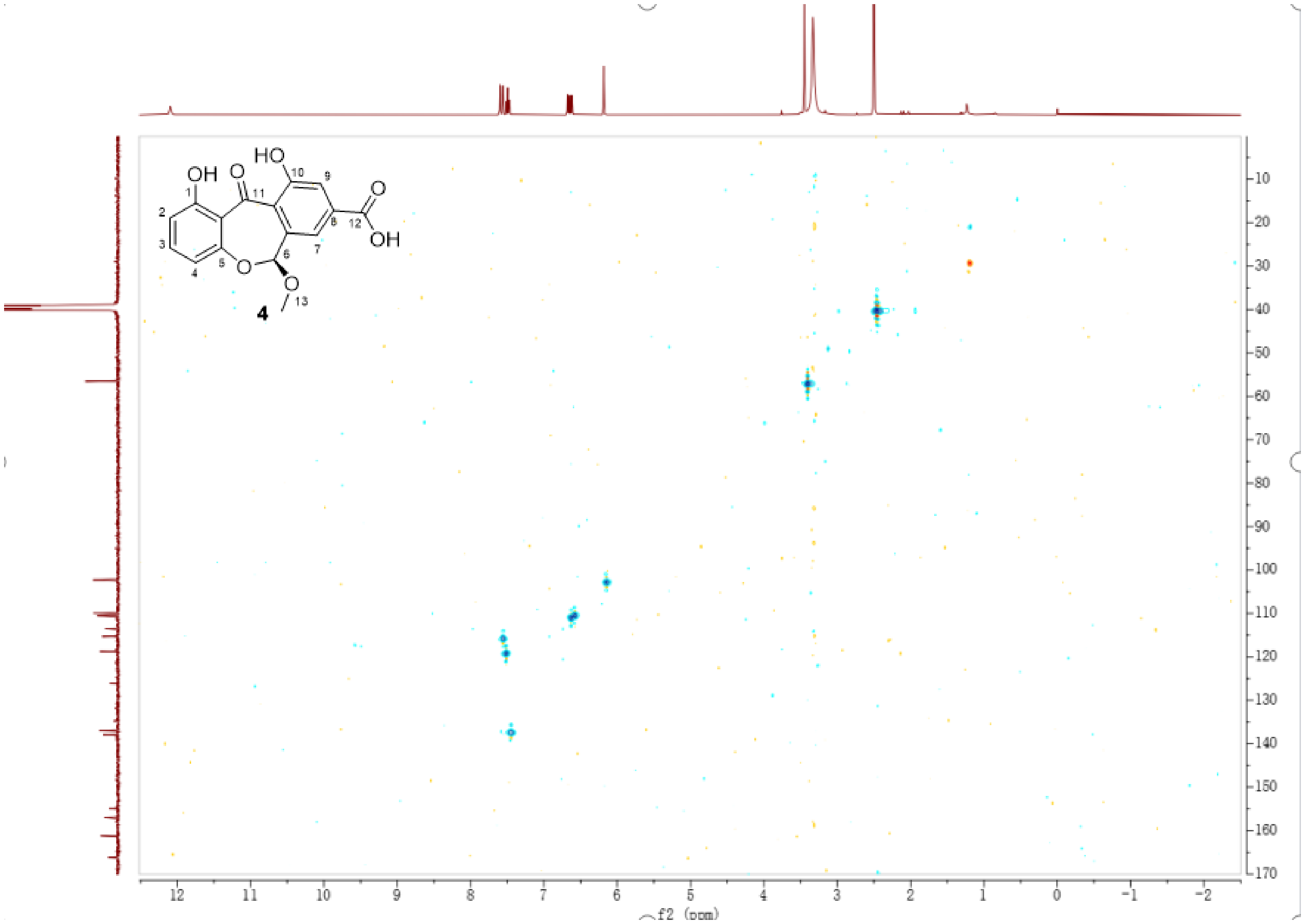

Figure S33. HMBC spectrum of compound **4**.

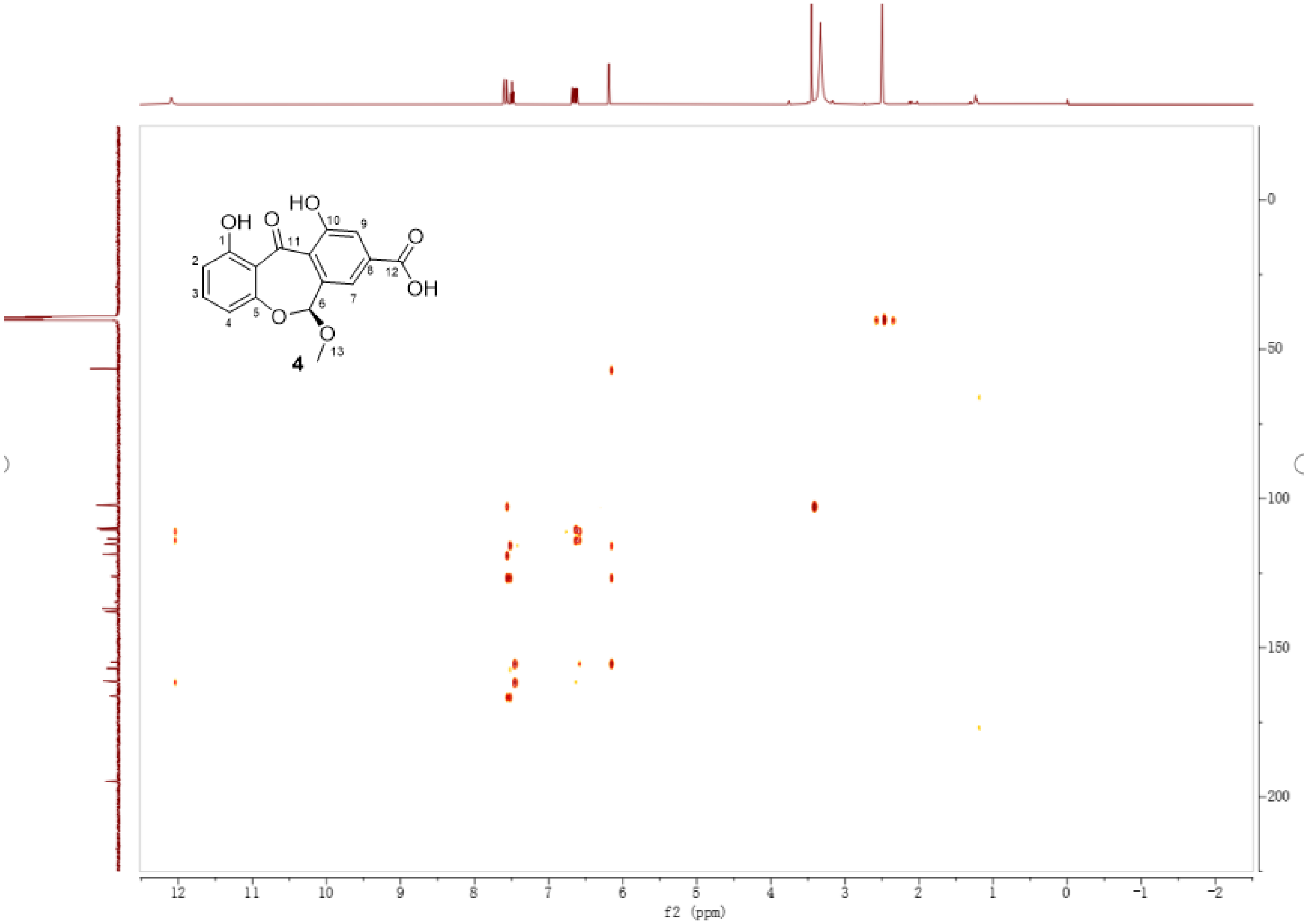

Figure S34. UV spectrum of compound **4**.

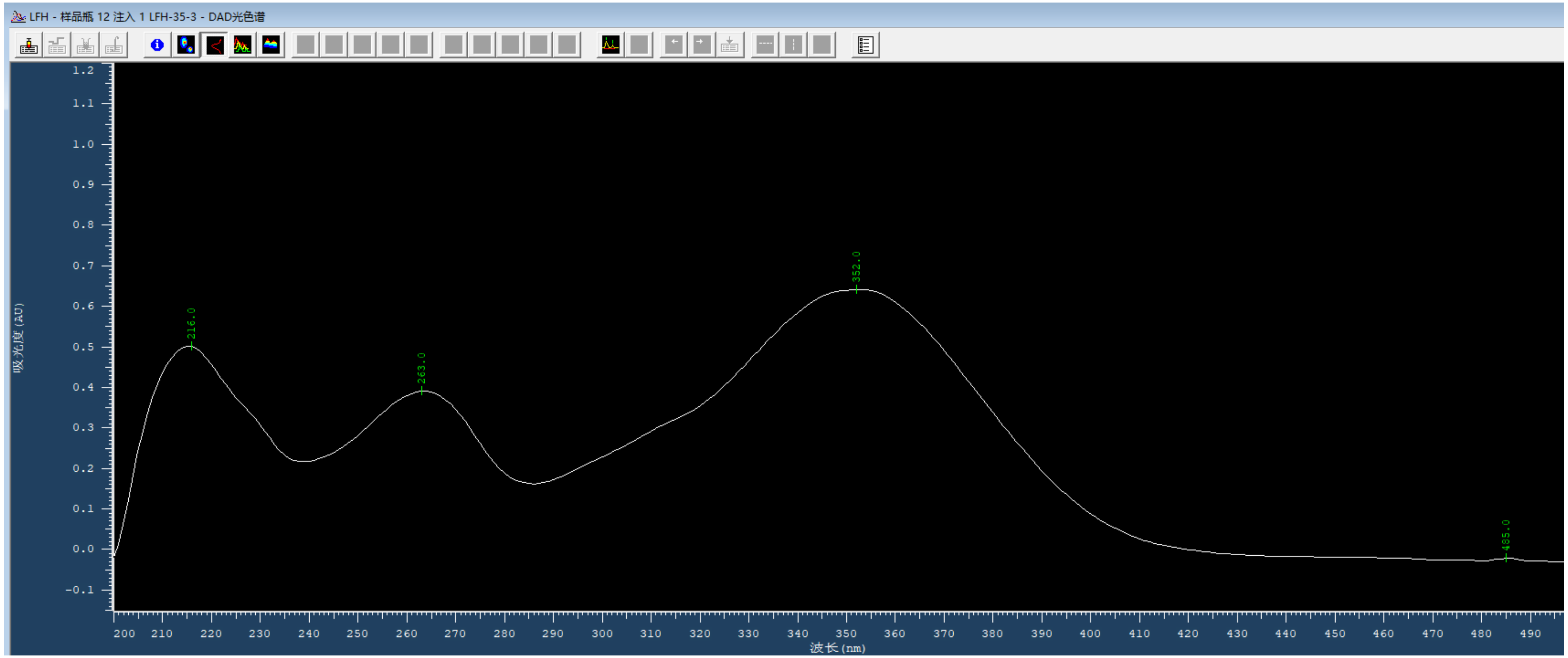

Figure S35. IR spectrum of compound **4**.

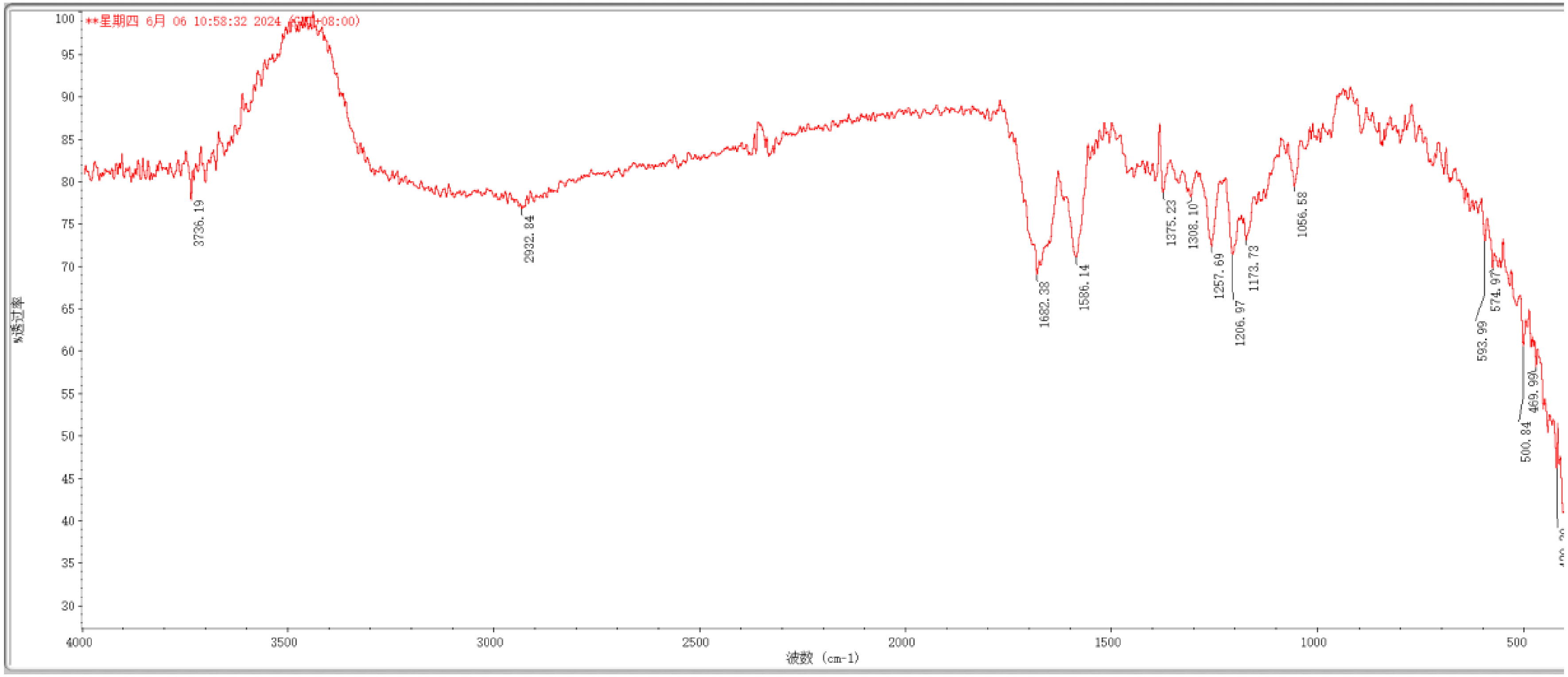

Figure S36. ^{1}H NMR (400 MHz, MeOD-d_4) spectrum of compound **5**;

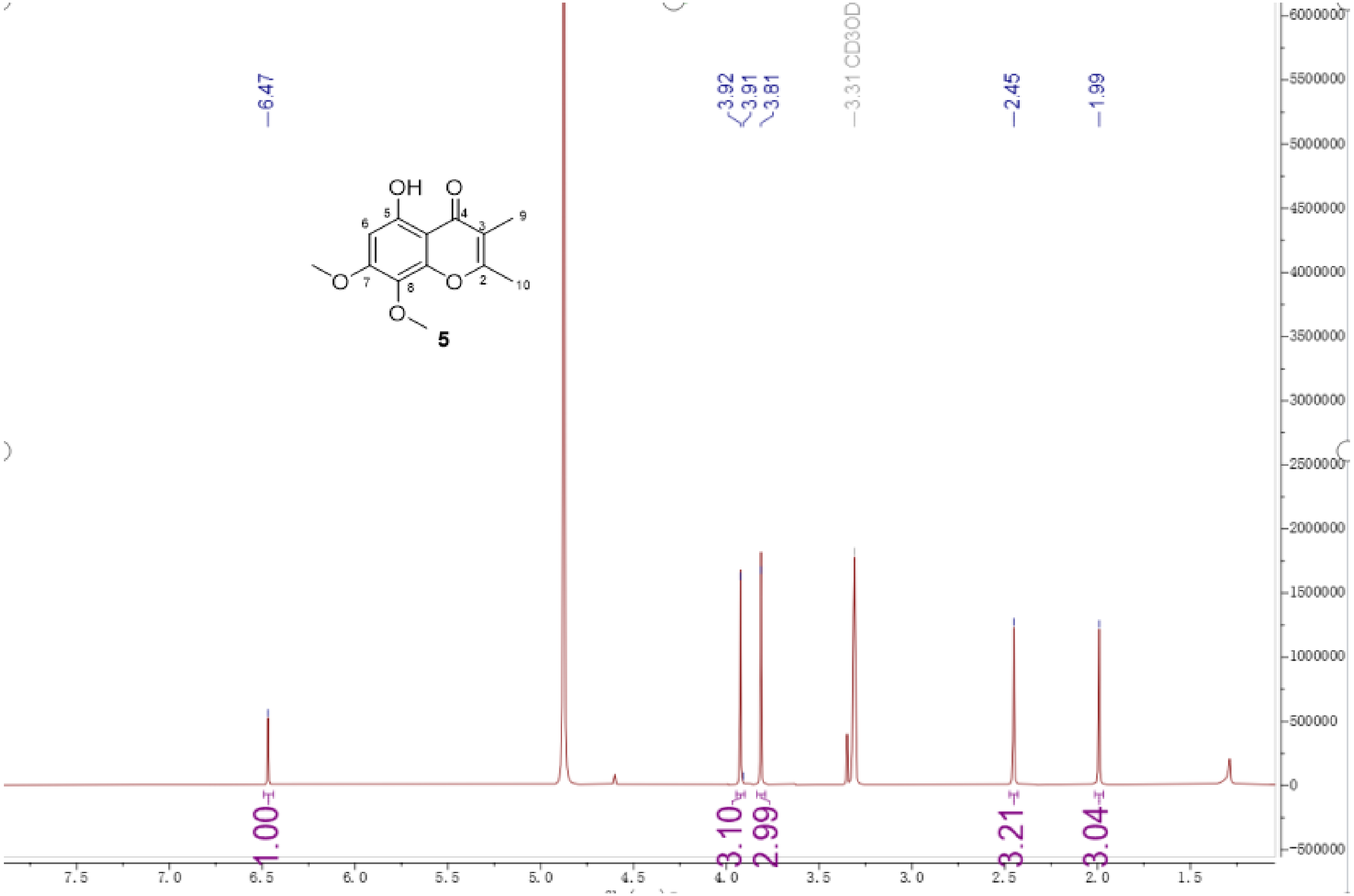

Figure S37. ^{13}C NMR (150 MHz, MeOD-d_4) spectra of compound **5**;

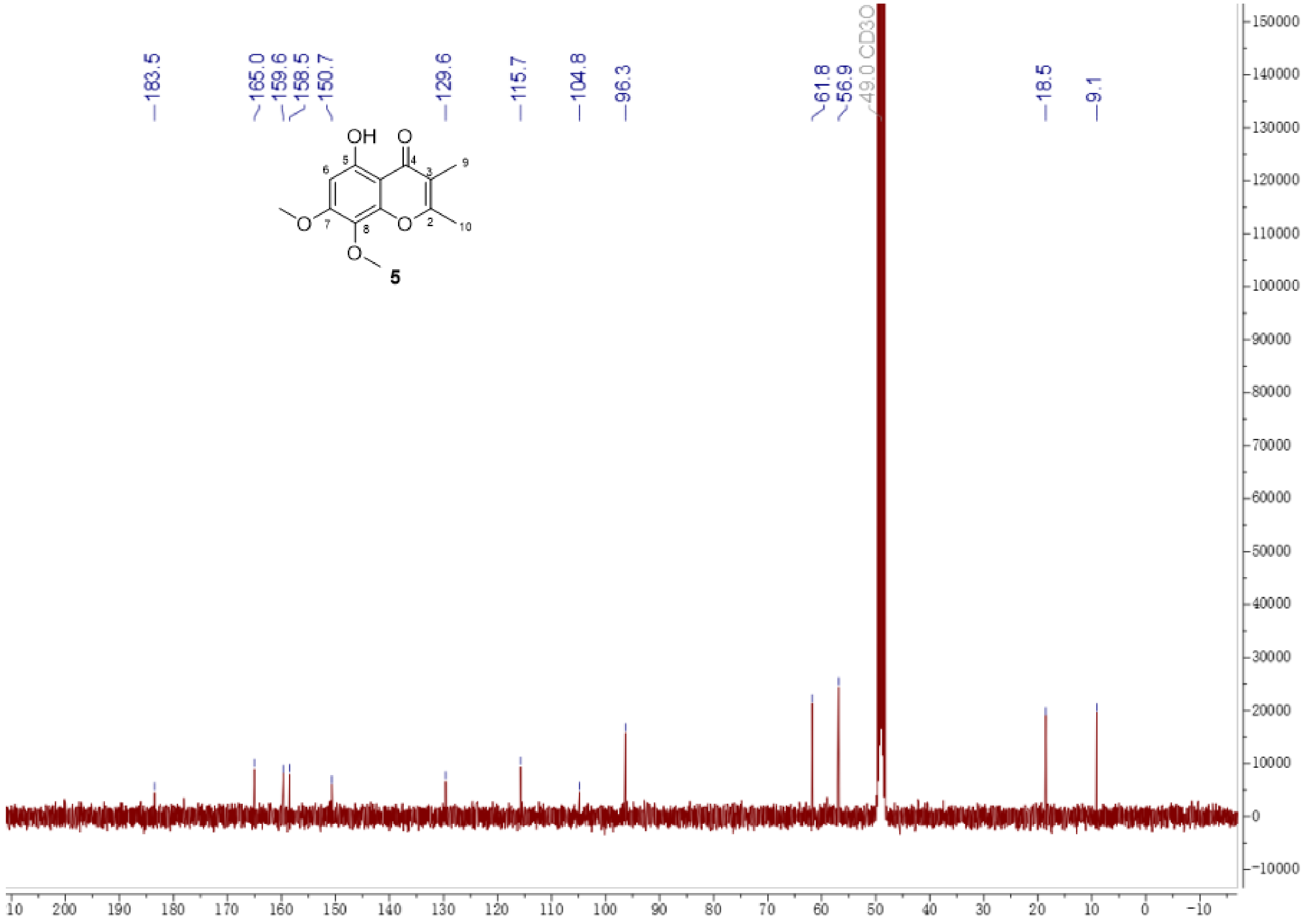

Figure S38. ^{1}H NMR (400 MHz, DMSO-d_6) spectrum of compound **5**;

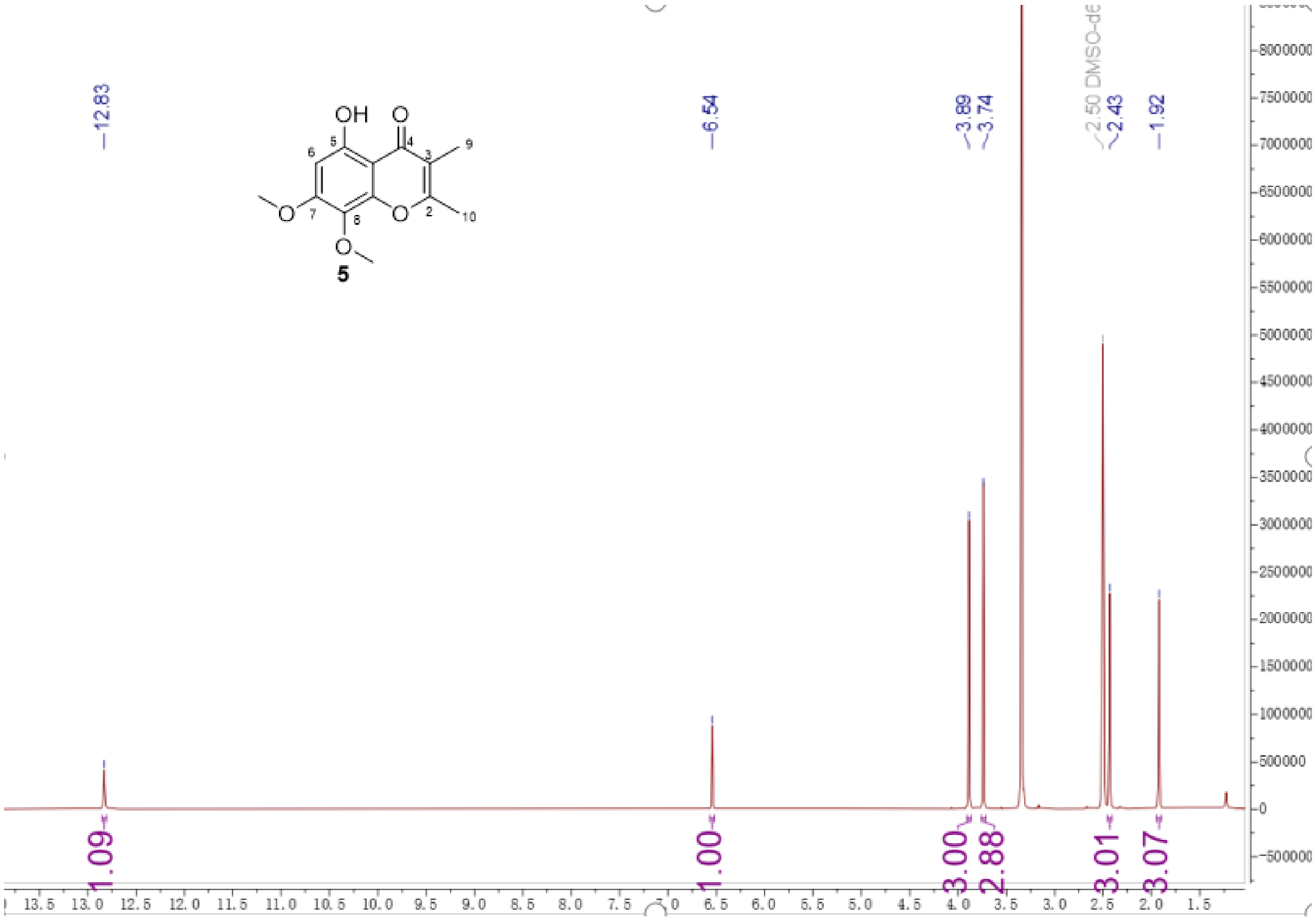

Figure S39. ^{13}C NMR (150 MHz, DMSO-d_6) spectra of compound **5**;

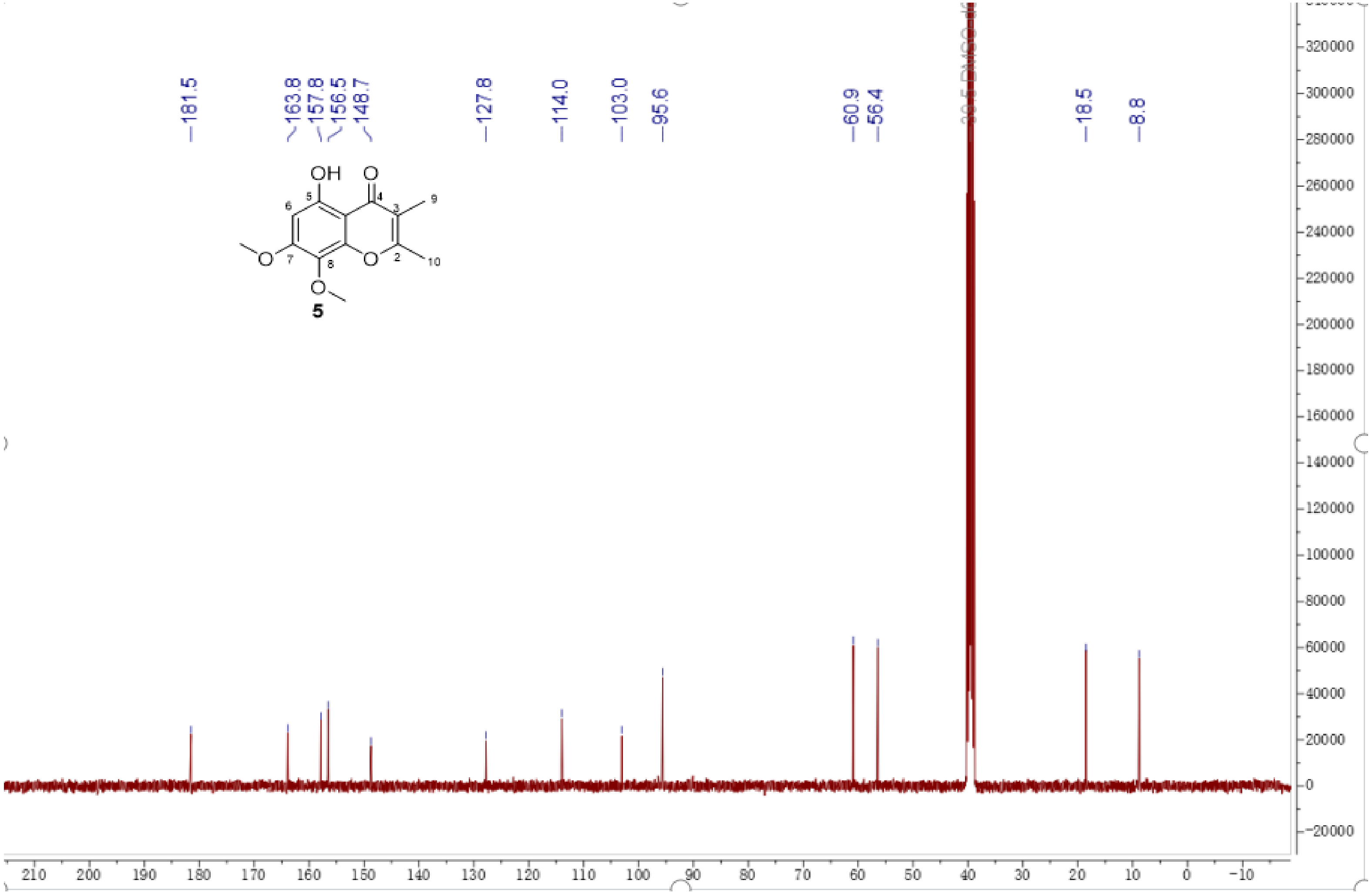

Figure S40. ^{1}H NMR (400 MHz, CD_3OD-*d*4) spectrum of compound **6**;

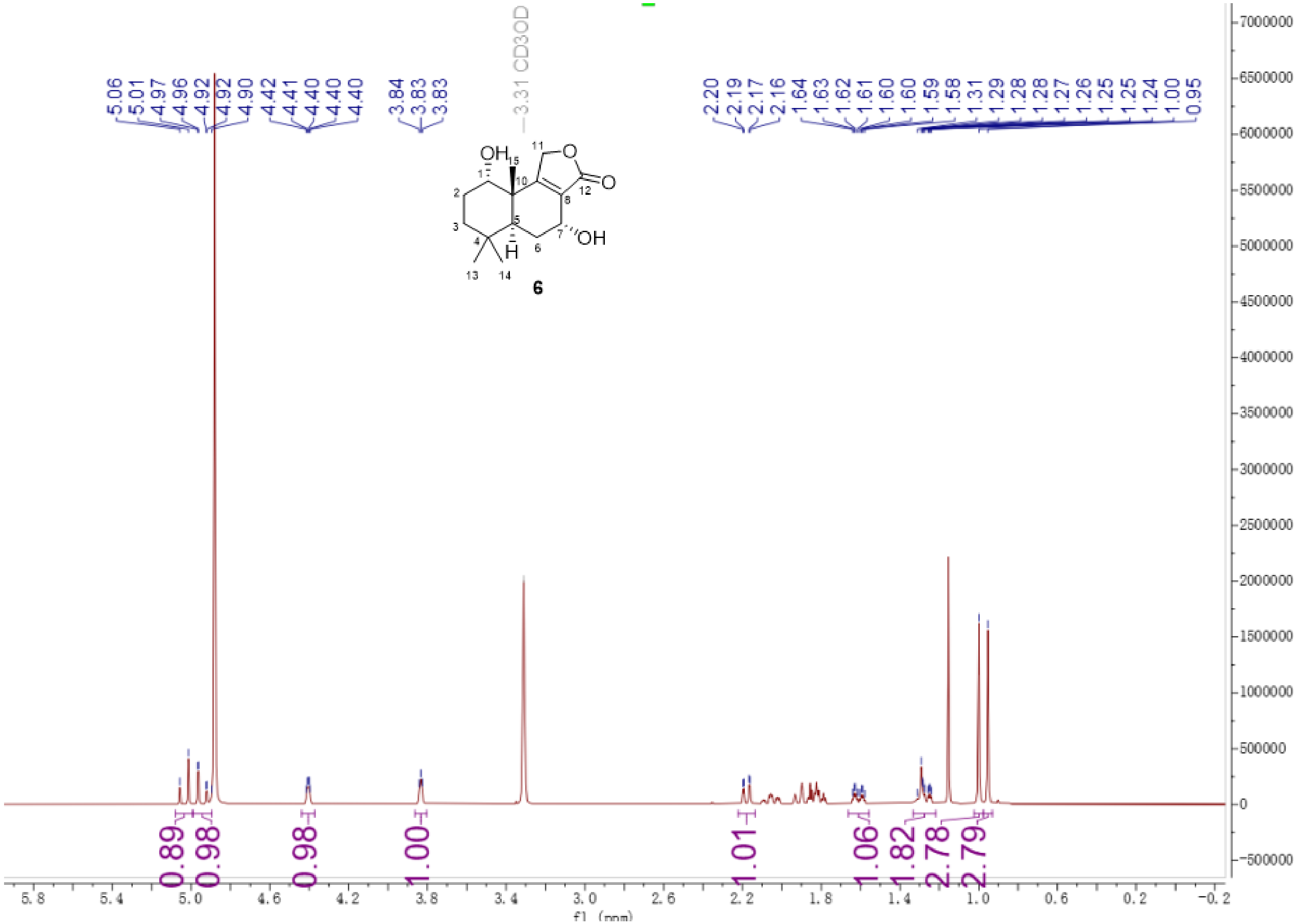

Figure S41. ^{13}C NMR (150 MHz, CD_3OD-*d*[4]) spectra of compound **6.**

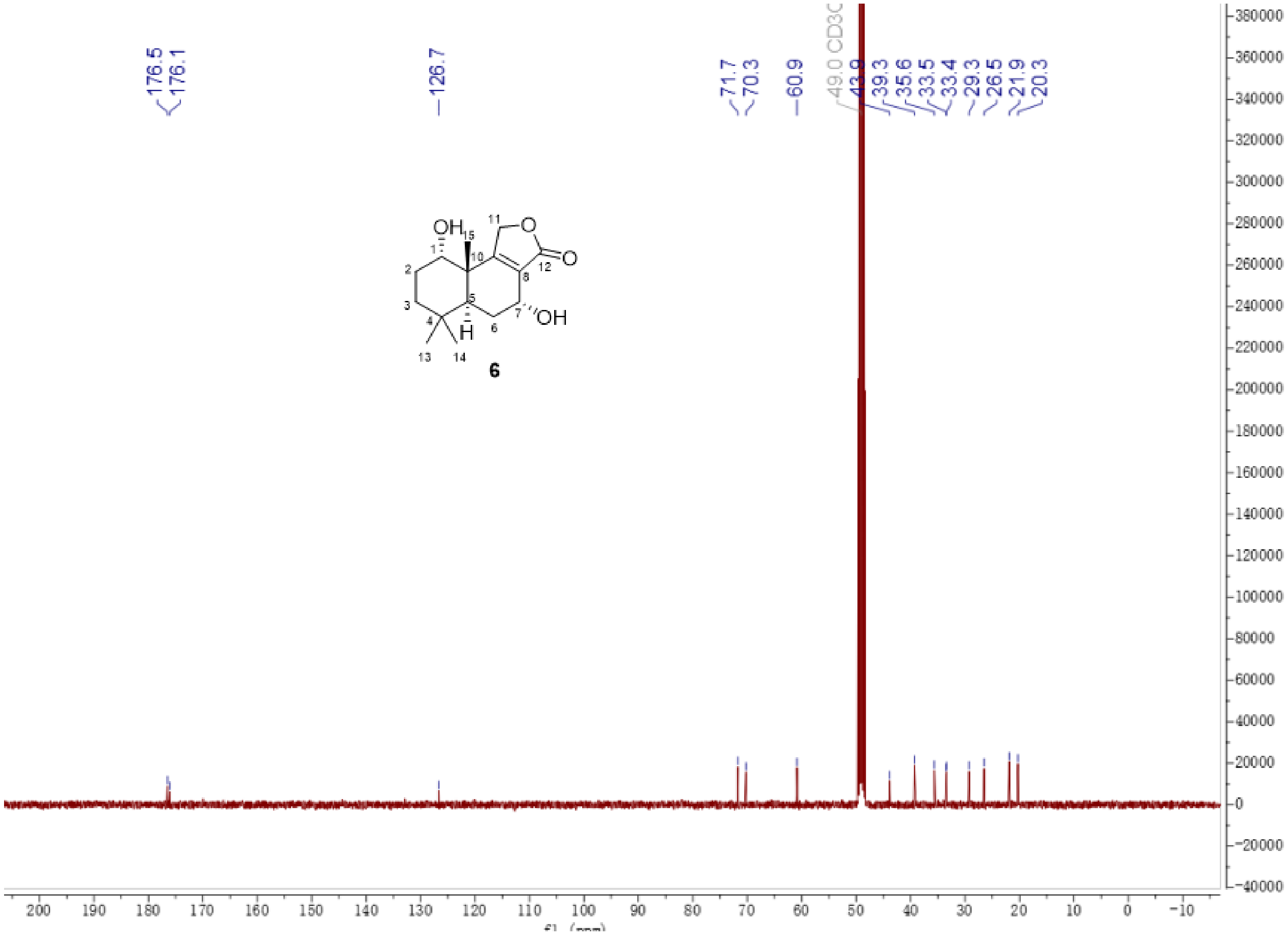

Table S1. The comparison of NMR data between compound **5** in DMSO-d_6 and talamin B in DMSO-d_6.

No.	5	Talamin B	5	Talamin B
	δ_H	δ_H	δ_C, Type	δ_C, Type
2			163,8, C	163.8, C
3			114,0, C	114.0, C
4			181.4, C	181.4, C
4a			103.0, C	103.0, C
5			157.8, C	157.8, C
6	6.54, s	6.53, s	95.5, C	95.5, C
7			156.5, C	156.5, C
8			127.8, C	127.8, C
8a			148.7, C	148.7, C
9	1.92, s	1.90, s	8.8, CH_3	8.8, CH_3
10	2.43, s	2.40, s	18.5, CH_3	18.5, CH_3
11	3.89, s	3.88, s	60.9, CH_3	60.9, CH_3
12	3.75, s	3.74, s	56.4, CH_3	56.4, CH_3

Table S2. The comparison of NMR data between compound **6** in CD_3OD-*d₄* and astalaminoid C in $CDCl_3$-*d*.

No.	6	astalaminoid C	6	astalaminoid C
	δ_H	δ_H	δ_C, Type	δ_C, Type
1	3.83, t	3.86, brs	71.7, CH	71.6, CH
2	1.61, m<br>2.05, m	1.65, m<br>2.07, m	26.5, CH_2	26.5, CH_2
3	1.25, m<br>1.82, m	1.31, m<br>1.82,m	35.6, CH_2	35.4, CH_2
4			33.5, C	33.3, C
5	2.17,dd	2.19, dd	39.3, CH	39.2, CH
6	1.82, m<br>1.90, dt	1.87, m<br>1.96, brd	29.3, CH_2	29.1, CH_2
7	4.40,dt	4.45, dd	60.9, CH	60.7, CH
8			126.7, C	126.6, C
9			176.1, C	175.9, C
10			43.9, C	43.7, C
11	4.94, dd<br>5.03, d	4.95, dd<br>5.08, brd	70.3, CH_2	70.1, CH_2
12			176.1, C	176.1, C
13	1.00, s	1.05, s	33.4, CH_3	33.3, CH_3
14	0.95, s	0.99, s	21.9, CH_3	21.9, CH_3
15	1.15, s	1.18, s	20.3, CH_3	20.4, CH_3
